# Risk factors and variations in detection of new bovine tuberculosis breakdowns via slaughterhouse surveillance in Great Britain

**DOI:** 10.1371/journal.pone.0198760

**Published:** 2018-06-08

**Authors:** Trevelyan J. McKinley, Debby Lipschutz-Powell, Andrew P. Mitchell, James L. N. Wood, Andrew J. K. Conlan

**Affiliations:** 1 College of Engineering, Mathematics and Physical Sciences, University of Exeter, Penryn, Cornwall, United Kingdom; 2 Disease Dynamics Unit, Department of Veterinary Medicine, University of Cambridge, Cambridge, Cambridgeshire, United Kingdom; 3 Data Systems Group, Animal and Plant Health Agency, Weybridge, Surrey, United Kingdom; Wageningen Universiteit, NETHERLANDS

## Abstract

Slaughterhouse surveillance through post-mortem meat inspection provides an important mechanism for detecting bovine tuberculosis (bTB) infections in cattle herds in Great Britain (GB), complementary to the live animal skin test based programme. We explore patterns in the numbers of herd breakdowns detected through slaughterhouse surveillance and develop a Bayesian hierarchical regression model to assess the associations of animal-level factors with the odds of an infected animal being detected in the slaughterhouse, allowing us to highlight slaughterhouses that show atypical patterns of detection. The analyses demonstrate that the numbers and proportions of breakdowns detected in slaughterhouses increased in GB over the period of study (1998–2013). The odds of an animal being a slaughterhouse case was strongly associated with the region of the country that the animal spent most of its life, with animals living in high-frequency testing areas of England having on average 21 times the odds of detection compared to animals living in Scotland. There was also a strong effect of age, with animals slaughtered at > 60 months of age having 5.3 times the odds of detection compared to animals slaughtered between 0–18 months of age. Smaller effects were observed for cattle having spent time on farms with a history of bTB, quarter of the year that the animal was slaughtered, movement and test history. Over-and-above these risks, the odds of detection increased by a factor of 1.1 for each year of the study. After adjustment for these variables, there were additional variations in risk between slaughterhouses and breed. Our framework has been adopted into the routine annual surveillance reporting carried out by the Animal Plant Health Agency and may be used to target more detailed investigation of meat inspection practices.

## Introduction

Bovine tuberculosis (bTB) is the most economically important disease of livestock currently affecting cattle in Great Britain (GB), estimated to cost the UK government >£100 million per year in direct costs [1]. Slaughterhouse surveillance of cattle, where every animal slaughtered is examined for signs of disease, is an essential part of the control program in GB, particularly in low-risk areas. Historically, the proportion of bTB incidents disclosed at the slaughterhouse has been lower in GB than other high-risk countries such as Ireland [2]. A recent increase in this proportion [3] is of concern to policy makers, as it may equally well reflect a reduction in the effectiveness of skin testing as an increase in the effectiveness of slaughterhouse surveillance. The rates of bTB submissions from different slaughterhouses vary considerably, but will depend on various factors including the effectiveness of meat inspection but also the risk profile of the input population. In this paper we explore these patterns in detail and identify risk factors for the disclosure of bTB incidents in herds through slaughterhouse tracebacks. However, our primary aim is to develop a robust statistical tool that can identify atypical slaughterhouses for further investigation, to contribute towards a better understanding of the effectiveness of slaughterhouse surveillance in GB.

Central to the statutory program of bTB control in GB is the mandatory skin testing of herds, using the single intradermal comparative cervical tuberculin (SICCT) test. Traditionally, herds were tested every 1–4 years depending on their *parish testing interval* (PTIs 1, 2, 3 and 4 respectively). Since 2013, the frequency of testing is now determined at the county, rather than parish level. South West England is classified as a high-risk area (annually tested during the study period) and is surrounded by an “edge area” (also annually tested during the study period). The rest of the country—the low-risk area—is generally on 4-yearly testing. Wales is entirely on annual testing and Scotland wholly on 4-yearly testing. A current spatial map of testing areas can be found in [4].

The SICCT test, although highly specific (> 99.9%) [5], has a moderate estimated sensitivity with respect to post-mortem confirmation (≈ 75–95.5%) [6, 7]. Furthermore, due to many factors, including animal movements and the frequency of herd testing, some animals are never tested in their lifetime [8], particularly in low-frequency testing areas. Post-mortem meat inspection therefore provides an important second arm of the surveillance system, particularly in low-risk areas where only breeding animals are subject to testing. When lesions are disclosed, movement restrictions are imposed on the herd of origin and a whole herd test (a check test) is scheduled. A *breakdown* is triggered by the disclosure of skin-test positive animals or through confirmation of *M. bovis* infection in slaughterhouse cases by bacteriological culture. Breakdown herds are then required to clear a fixed number of whole herd tests before restrictions are lifted. The number of tests a herd must pass to regain officially TB free status depends on whether any reactors in the breakdown have been confirmed (either by characteristic gross TB lesions or culture) or not. Breakdowns with no confirmed reactors are described as “Officially TB Free Suspended” (OTFS) and historically were only required to pass a single clear test for movement restrictions to be lifted. More recently these conditions have changed, and from January 2014 all bTB breakdown herds in the edge area must pass two herd tests with negative results at a more severe interpretation of the test. From April 2016 this has been extended to all breakdown herds in the high-risk area. Confirmation of any reactors or lesions found at routine slaughter leads to a classification of “Officially TB Free Withdrawn” (OTFW) where the herd must clear two consecutive herd tests at the severe interpretation.

This dynamic link between slaughterhouse surveillance, SICCT testing and post-mortem confirmation means that neither arm of the system can be considered independent. Deficiencies in skin testing will manifest in increased rates of disclosure of lesioned animals at the slaughterhouse. Likewise, missing lesioned animals at the slaughterhouse potentially increases the time that undisclosed transmission can occur within herds. Although the sensitivity of slaughterhouse detection is generally believed to be low, its overall effectiveness in GB is manifest in the consistency of the distribution of reactor animals removed from breakdowns in different risk areas [9], and in its critical role in Scotland and low-risk areas of England.

In the Republic of Ireland (ROI), separate logistic regression models were estimated for the individual animal risk of submission of suspect lesions and laboratory confirmation [10, 11]. The goal of these Irish studies was to provide separate (ranked) estimates for the rates of submission of suspect lesions and laboratory confirmation per slaughterhouse respectively, after adjusting for potential confounding risk factors in the input population of each slaughterhouse. These studies estimated the impacts of individual- and herd-level effects of age, sex, season and indirect measures of exposure to bTB (e.g. risk class, number of years clear of restrictions, whether animal was homebred), along with a fixed effect representing the relative difference between each slaughterhouse. In general the individual level risk of both submission and confirmation was found to increase with age and risk of exposure, with a slightly increased risk of confirmation for female animals (which could be confounded with differences between dairy and beef management practices).

Similar research was conducted in Northern Ireland (NI) [12], again focusing on risk factors for disclosure of bTB in slaughterhouses, and for the probability of confirmation. This study looked at the effects of age, sex, season, patch incidence (e.g. risk region), past history of bTB on the disclosing herd and whether or not the animal was homebred or purchased. In general the risk of disclosure increased with age and for animals sourced from high-risk areas, with a higher risk of disclosure in females, purchased animals and animals slaughtered in winter. There was a slightly more complex relationship with herd history. We note that in ROI and NI the number of slaughterhouses (42 and 10 respectively) is much smaller than in GB (276).

In GB, an alternative approach was used to model the risk of detecting *M. bovis* in samples from lesions found in cattle that were consistent with being infected; quantifying the variability between slaughterhouses using a random intercept term [13]. Their study population consisted only of animals that had been sent to slaughter from Officially TB Free (OTF) herds, and individual level factors adjusted for by the model included year of birth (rather than age), year of slaughter (2003–2008) and further indirect measures of risk of exposure to bTB (e.g. region of farm of origin of bovine, parish testing interval of farm of origin, and number of reactors in most recent previous breakdown on farm). In common with the Irish studies, the risk of confirmation increased with indirect measures of exposure and a large degree of variability in confirmation rates between slaughterhouses was reported, ranging from 21–81%, although this could reflect varying prevalence of other, confounding, intercurrent disease. In their final model the probability of confirmation was found to decrease over time with year of slaughter, but increase with date-of-birth.

In this manuscript we tackle a slightly different question and quantify the contribution that slaughterhouses make to the overall detection rate of bTB breakdowns in GB, rather than the rate of submission or confirmation of individual animals. To this end we define our cases as all animals with suspicious lesions submitted for laboratory confirmation that were either subsequently confirmed, or that triggered the disclosure of new skin test reactor animals in the originating herd. Our case definition therefore captures the overall effectiveness of slaughterhouse surveillance at the herd, rather than the individual animal level. In addition to hierarchical terms that estimate the variability between slaughterhouses, we also include an additional hierarchical term to account for possible confounding effects between breeds in terms of susceptibility [14], reaction to diagnostic tests and post-mortem confirmation [15].

## Materials and methods

Bovine TB is a notifiable infectious disease in GB. Surveillance data recording the results of all testing and slaughterhouse cases are collated by the Animal and Plant Health Agency (APHA) in the Sam database and linked with cattle movement records from the Cattle Tracing System (CTS). Data downloads from the Sam system were used to construct local databases using PostgreSQL (https://www.postgresql.org/) with subsequent analysis and modelling carried out using the open-source statistical language R [16].

### Number of breakdowns per herd over time

The numbers of OTFS and OTFW breakdowns identified between 1998–2013 were extracted, along with the associated disclosing test responsible for initiating each breakdown (55,653 entries). Data where first tests were missing or unknown, or ‘queried’ data (entered as ‘VE-QSLH’) were removed (39 entries), leaving 55,614 entries.

### Risk factors for identifying a breakdown through routine slaughterhouse surveillance

All slaughterhouse (SLH) cases that disclosed a breakdown and submitted between 2004 and 2013 for laboratory confirmation were extracted as our study population. This period was chosen to avoid bias resulting from the well described impact of the suspension of SICCT testing during the 2001 foot-and-mouth epidemic [2]. Since one of the principal aims of the study was to quantify differences between slaughterhouse detection rates, over-and-above the background risk of detection based on individual-level risk factors, it was necessary to sample controls *proportionately* between slaughterhouses. This ensures that we are able to obtain unbiased estimates of the odds ratios for each slaughterhouse, relative to an average slaughterhouse (see model specification below). In addition, since there is considerable variability in the throughput of slaughterhouses, a simpler, non-stratified random sample would have resulted in some slaughterhouses having either no, or very few, controls. Since we were using all the available cases, the overall statistical power can also be improved by sampling disproportionately from the control population. Hence we extracted a stratified random sample of 0.3% of skin test negative animals with no visible lesions at slaughter from each slaughterhouse as our control population. This ensured reasonable coverage within each slaughterhouse and that the model was computationally manageable. After records with other missing data were removed, we ended up with 77,426 samples, consisting of 8,607 cases and 68,819 controls. Summaries of the raw data can be found in the S1 File, and a copy of the raw data can be found in the S1 Data file.

Risk was modelled at the individual animal level using a range of explanatory factors (Table 1) that capture the temporal and individual variations in risk of exposure and presentation at slaughterhouses. In common with previous studies: sex, seasonal quarter, region and age are included as baseline risk factors [10–13]. Through linking the Sam and CTS databases a range of alternative indirect measures of exposure were calculated for each animal that capture different aspects of the lifetime risk-of-exposure for each animal.

**Table 1:** Animal-level variables used in regression model.

Variable	Description	Type	Values
Sex	Sex of animal	Categorical	F / M
Year	Year post 2003 that animal was slaughtered	Integer	1-10
Quarter	Quarter that animal was slaughtered	Categorical	Jan-Mar,…, Oct-Dec
Risk region	Region animal was present for most of its life	Categorical	HFT (PTI 1 & 2) in England / Wales LFT (PTI 3 & 4) in England/ Scotland / Wales
Movement indicator	No. of movements between herds	Categorical	0, ≥ 1
Age	Age at slaughter (months)	Categorical	(0, 18], (18, 24], (24, 36], (36, 60], >60
Contact in low-risk herds	Contact days in low-risk herds	Categorical	None / Some
Contact in high-risk herds	Contact days in high-risk herds	Categorical	None / Some
Number of herd tests present for	Number of herd tests an animal is present for	Categorical	0, 1, >1
SLH	Slaughterhouse indicator (where the animal was slaughtered)	Categorical	276 levels
Breed	Breed	Categorical	166 levels
SLH case	SLH case	Binary	0 / 1 (no / yes)

The simplest of these is the number of lifetime herd-to-herd movements. This factor distinguishes the differential risk of exposure between cattle that remain in the same herd in their lifetime (no moves) or are traded (one or more moves). We aim to approximate the duration of time that an animal may have been exposed to bTB through the number of days spent in a high-risk herd. A previous history of bTB is a consistent risk factor for predicting future breakdowns [9, 13, 17, 18]. We choose to define high-risk herds to be any herd with a history of bTB restrictions within the 5 years previous to the date of the slaughterhouse disclosure. In contrast, low-risk herds are defined as those with no reported bTB incidents in the 5 years prior to disclosure. The duration distributions are heavy-tailed, and after taking log(*duration*+ 1) transformations, the duration of time in high-risk herds (in particular) is strongly bimodal (Fig A in S1 File). Hence for simplicity we decided to simplify this measure into two categorical variables: the first describes the duration of stays on low-risk herds as “None” or “Some”, and the second is similar but for stays on high-risk herds. Finally, the number of herd tests that an animal was present for is included as a proxy measure for both the risk of transmission within the herd, but also the likelihood that an infected animal could be identified by SICCT testing before slaughter.

To specify the model, we define the binary response variable:

$$Y_{i}=\{\begin{array}{cc} 1 & \text{if}\;\text{animal}\;i\;\text{is}\;\text{SLH}\;\text{case},\\ 0 & \text{otherwise}. \end{array}$$ (1)

and assume *Y*_*i*_ ∼ Bernoulli(*p*_*i*_). Here,

$$\text{log}(\frac{p_{i}}{1-p_{i}})=\beta^{T}x_{i}+\theta_{B_{i}}+\gamma_{S_{i}},$$ (2)

where the **x**_*i*_ are a vector of explanatory variables (shown in Table 1), and *B*_*i*_ and *S*_*i*_ correspond to the breed and slaughterhouse for individual *i* (*i* = 1, …, *N*). The *J* + 1 regression parameters are given by *β* = {*β*_*j*_; *j* = 0,…,*J*}, and *θ*_*k*_ and *γ*_*l*_ are the hierarchical terms (analogous to a random intercepts model), where *k* = 1, …, *N*_*B*_ and *l* = 1, …, *N*_*S*_, with *N*_*B*_ = 166 and *N*_*S*_ = 276 being the number of breeds and slaughterhouses respectively. (Summaries of the distributions of samples within each SLH and breed can be found in the S1 File).

Heterogeneity in slaughterhouse detection rates is modelled through the estimation of slaughterhouse-level hierarchical intercepts. This approach was taken to allow for the highly variable throughput of each SLH, with some slaughterhouses slaughtering many more animals than others. This results in a *shrinkage* model, where SLHs with low throughput will be shrunk towards the overall mean, thus de-emphasising the intercept term for slaughterhouses with low throughput (see e.g. [19]).

We formulated the model in a Bayesian framework, which allows full posterior distributions for the parameters to be obtained, as well as full posterior predictive distributions for various measures of interest. Due to the size of the data set under study we wrote our own Markov chain Monte Carlo (MCMC) code that allowed for parallelisation of the likelihood function to improve efficiency. We also increased efficiency by using a mix of centred and non-centered re-parameterisations [20]—see S1 File for full details. For ease of reproducibility, the code has been compiled into an R package called BayesLog, the current version of which can be found at https://github.com/tjmckinley/BayesLog. A link to the data can be found next to the published manuscript online, and the R code necessary to run the models can be found in the S1 File.

To complete the Bayesian specification, we set the prior distributions as:

$$\begin{array}{ccc} \beta_{j} & ∼ & N(0,100^{2}),\\ \theta_{k} & ∼ & N(0,\sigma_{\theta }^{2}),\\ \gamma_{l} & ∼ & N(0,\sigma_{\gamma }^{2}),\\ \sigma_{\theta } & ∼ & U(0,20),\\ \sigma_{\gamma } & ∼ & U(0,20). \end{array}$$

We ran two models: both including and excluding the slaughterhouse terms. Each model was run for two chains of 10,000 iterations each, with the first 5,000 samples from each chain discarded as burn-in. Convergence was assessed using visual inspection of MCMC trace plots. The fit of both models was examined in two ways: by using binned residual plots [21], and by exploring the goodness-of-fit using ROC-curve type analyses [22]. The goodness-of-fit measures were generated from 1,000 posterior samples, chosen at random. All results are reported to 2 significant figures.

## Results and discussion

### Number of breakdowns over time

**Fig 1:**
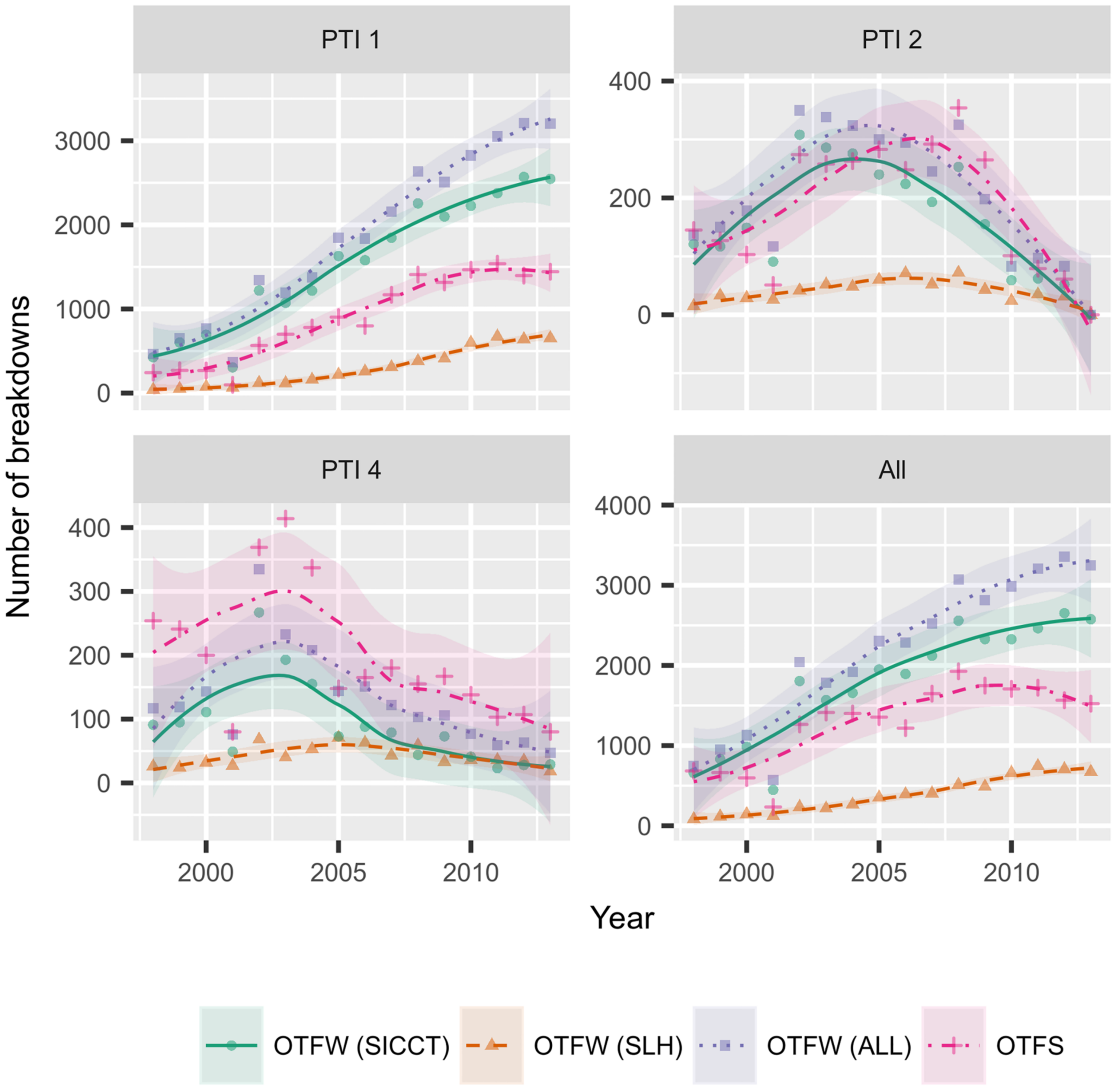
Descriptive plots of numbers of breakdowns of different types over time. Number of Officially TB Free Suspended (OTFS) and Officially TB Free Withdrawn (OTFW) breakdowns, and the number of OTFW breakdowns initiated either through slaughterhouse surveillance (SLH) or skin (SICCT) testing per year, stratified by Parish Testing Interval (PTI). (Note the different *y*-axis scales in each plot). The trend lines and 95% confidence intervals are obtained from simple loess smoothing.

We first explore the contribution of slaughterhouse surveillance to the number of bTB breakdowns stratified by the parish testing interval (PTI) as a proxy measure of risk. (Note that we exclude the rare historical PTI 3 herds in these plots. The uncommon PTI 2 areas were abolished in January 2013, with herds now classified as being in high-risk (PTI 1) or low-risk (PTI 4) regions [4]). Fig 1 compares the number of OTFS breakdowns to the number of OTFW breakdowns initiated either through slaughterhouse surveillance or SICCT testing for each year.

**Fig 2:**
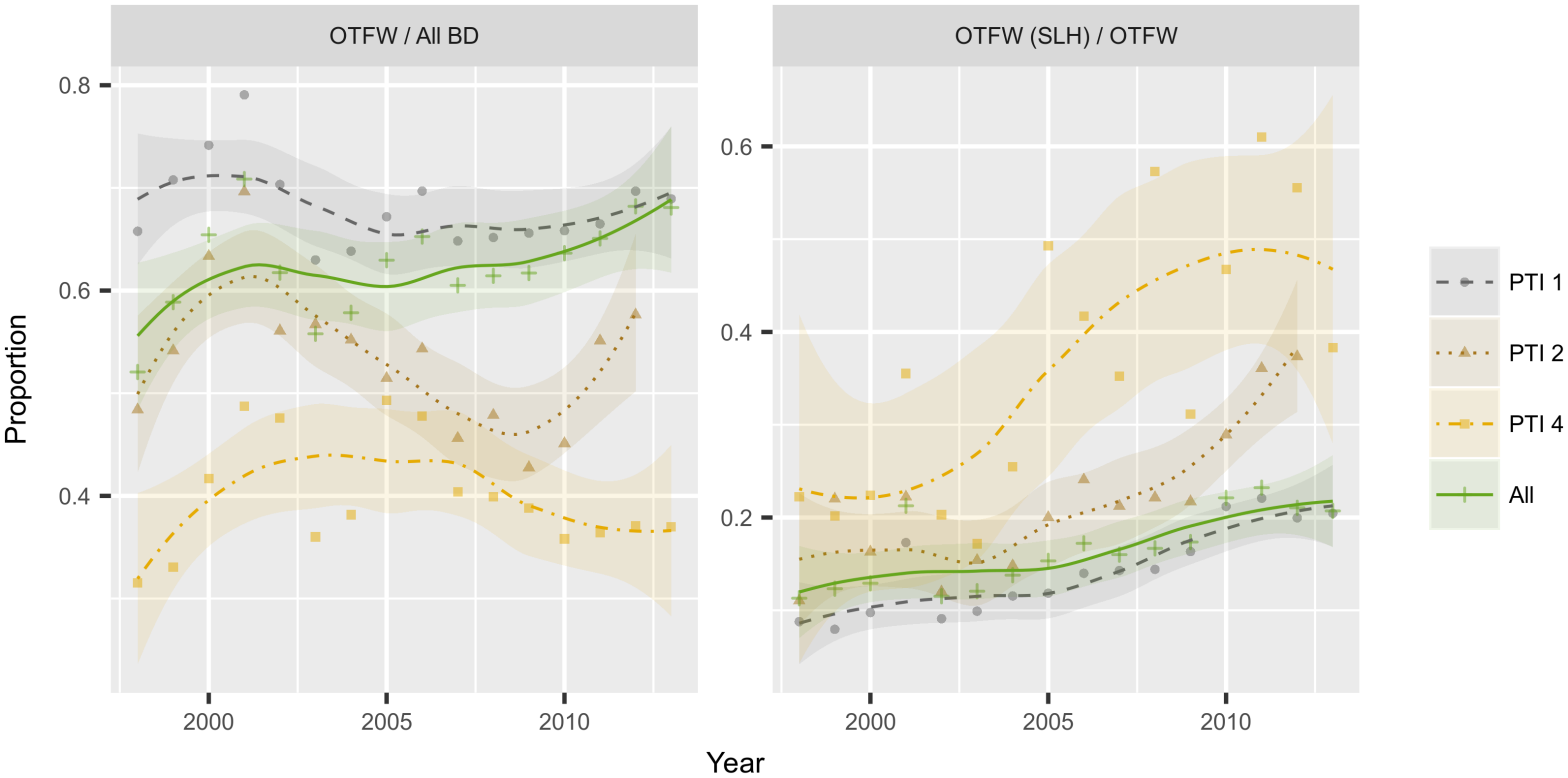
Descriptive plots of proportions of breakdowns of different types over time. Proportions of total breakdowns (BD) that are Officially TB Free Withdrawn (OTFW), and proportion of OTFW breakdowns that are initiated in the slaughterhouse (SLH) per year, stratified by Parish Testing Interval (PTI). (Note the different *y*-axis scales in each plot). The trend lines and 95% confidence intervals are obtained from simple loess smoothing.

The absolute number of breakdowns initiated in the slaughterhouse has increased across all three risk areas over the period of the study. However, this increase must be balanced against both the overall increase in the number of breakdowns over the period and the systematic shift of herds from the PTI 2 and 4 regions into PTI 1 accompanying this growing epidemic. This can be accounted for by considering the proportion of breakdowns initiated by different tests (Fig 2).

Overall, the proportions of total breakdowns that are OTFW are relatively stable, with some oscillation (Fig 2); as previously reported by APHA [3]. However, there was a continuing systematic increase in the proportion of OTFW breakdowns being initiated through SLH surveillance (Fig 2) across all risk areas.

A key feature of these data, although not directly related to slaughterhouse surveillance, is that there were proportionally more OTFS breakdowns in PTI 4 than in PTI 1, consistent with there being a lower positive predictive value of the screening skin test in low-risk areas compared to high-risk areas [5].

#### Logistic regression model fits

**Fig 3:**
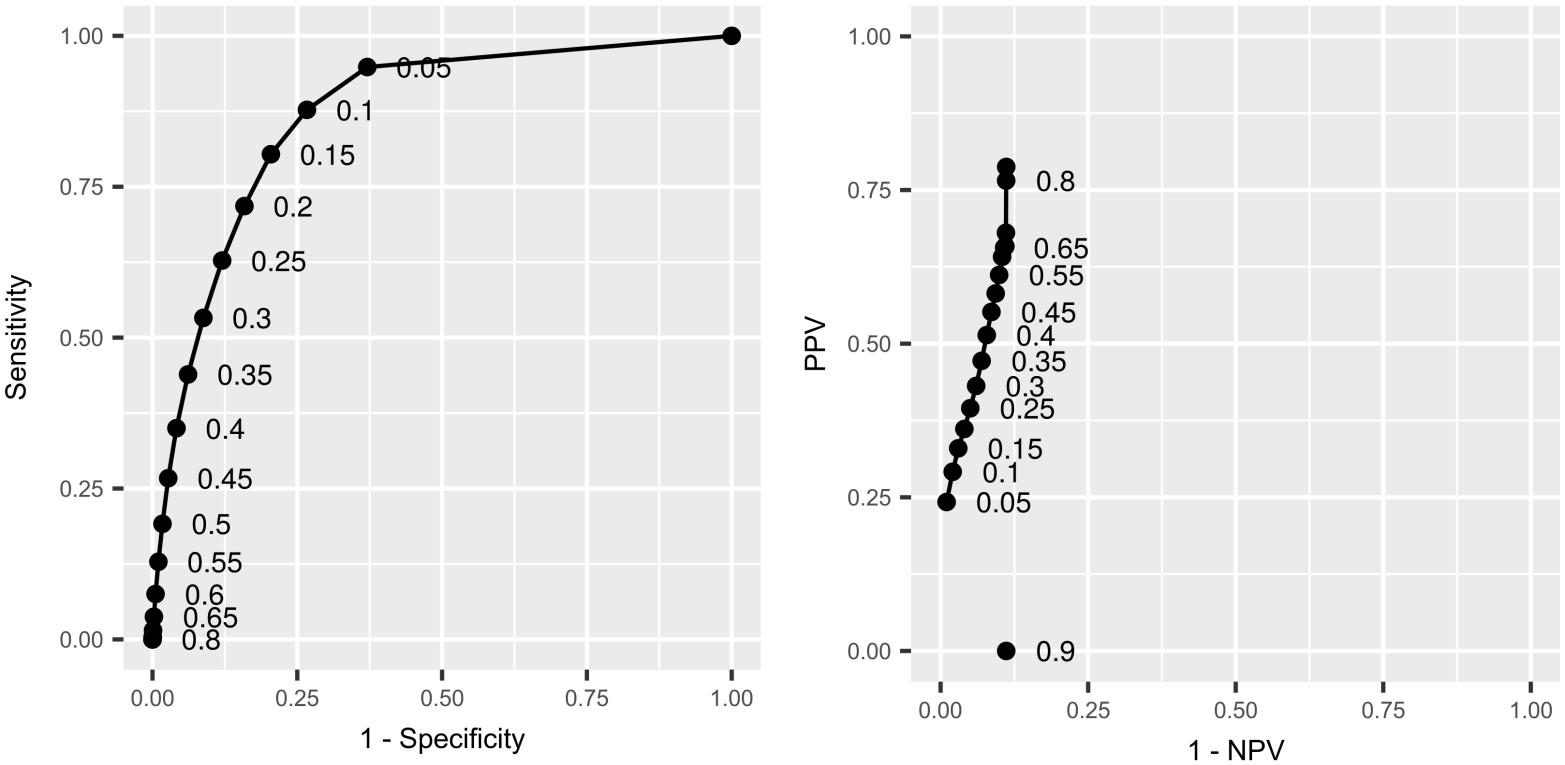
Classification curves. Receiver Operating Characteristic (ROC) and predictive value curves—positive predictive value (PPV) vs. negative predictive value (NPV)—for the model predictions relative to the observed data. Point estimates provided by the posterior predictive means.

Trace plots for both models are shown in the S1 File, and did not show any evidence for a lack-of-convergence. The binned residual plots show no systematic lack-of-fit (see Figs K and O S1 File). (We note some possible curvature in Fig O S1 File, but feel that the fit is still acceptable in this circumstance). The posterior predictive mean AUC (Area Under the ROC Curve) and 95% credible interval is 0.88 (0.88, 0.88) for the full model, and 0.85 (95% CI: 0.85–0.85) for the model without SLH effects. However, we must be careful here, since the data are unbalanced, and so for the full model we also generated a curve exploring the relationship between the positive and negative predictive values for a range of probability thresholds (analogous to the ROC curve—Fig 3). We can see that the specificity and negative predictive value (NPV) are always high, but there is more variation in the sensitivity and positive predictive value (PPV). This is to be expected when the ratio of controls to cases in the data is unbalanced in favour of controls. Nevertheless, there are probability thresholds that can find a good balance between these values (e.g. a probability threshold of *p* = 0.35 gives high NPV and specificity values, but also a sensitivity and PPV of around 45–55%—as shown in Figs E and F S1 File). Overall we conclude that the model fits well and is a good classifier for these data.

#### Risk factors

Odds ratios (ORs) for the explanatory variables obtained from the full model (including the SLH terms) are shown in Table 2. (For brevity we exclude the hierarchical terms from Table 2). Note that we have set the baseline level of each categorical variable to be the level with the lowest risk. This means that all ORs are positive, and can be directly compared on the same scale.

**Table 2:** Odds ratios for explanatory variables with 95% credible intervals.

Variable	Levels	Controls	Cases	OR	2.5%	97.5%
Sex	M	35446	3119	1		
	F	33373	5488	1.1	0.99	1.1
Year (per year)				1.1	1.1	1.1
Quarter	Apr—Jun	16522	1854	1		
	Jul—Sep	16605	2035	1.1	0.99	1.2
	Oct—Dec	18585	2668	1.2	1.1	1.3
	Jan—Mar	17107	2050	1.1	0.99	1.2
Risk Region	LFT Scotland	15803	53	1		
	LFT England	25111	992	6.6	4.5	9.2
	LFT Wales	3552	275	7.2	4.9	10
	HFT Wales	5552	912	12	8.4	17
	HFT England	18801	6375	21	15	29
Movement Index	≥1	52270	5958	1		
	0	16549	2649	1.2	1.1	1.2
Age (in months)	(0,18]	12107	397	1		
	(18,24]	16706	1166	2	1.7	2.2
	(24,36]	25540	3381	2.4	2.2	2.8
	(36,60]	3694	776	3.4	2.9	4
	>60	10772	2887	5.3	4.6	6.1
Contact in low-risk herds	None	2467	546	1		
	Some	66352	8061	1.1	0.98	1.2
Contact in high-risk herds	None	41907	1321	1		
	Some	26912	7286	2	1.9	2.2
Number of herd tests present for	0	22593	584	1		
	1	11472	375	1	0.88	1.2
	>1	34754	7648	1.2	1.1	1.3

For details of variables, see Table 1.

The strongest single risk factor is the region where animals spend most of their lives, with (as expected) animals that spend most of their lives in high-risk areas showing greater odds of being SLH cases than animals that spend most of their lives in low-risk areas. Scotland has the lowest odds, with LFT areas in England and Wales showing odds that are on average 6.6–7.2 times larger than in Scotland. Compared to Scottish animals, the odds of being a SLH case are on average 21 times higher for animals living mostly in the HFT region of England, and 12 times higher for animals living mostly in the HFT region of Wales. Qualitatively similar effects were observed for analogous risk region (patch) variables in earlier studies [10, 12, 13].

As expected, there is a strong increasing effect of age, such that older animals are more likely to be picked up as slaughterhouse cases than younger animals (the odds of animals >60 months old being SLH cases are on average 5.3 times higher than the odds for than animals (0, 18] months old). Again, this general increase of risk with age was also observed in earlier studies in ROI and NI [10, 12], and is likely a proxy for an increased probability of infection, and also an increased duration of infection (thus increasing the time for the development of visible lesions).

There also seems to be an increase in the odds of SLH detection over time, such that for each year that passes from the beginning of the study period, the odds of being a SLH case increase by 1.1 on average. On the surface this seems to conflict with an earlier study [13], but it is worth bearing in mind that in [13] the authors were modelling the probability of confirmation, rather than the probability of detection. Our results suggest that there are additional year-on-year changes in risk that are not being captured by the other risk factors, which is in line with the general trend observed in the descriptive analysis (shown in Figs 1 and 2).

There is also some impact of whether or not an animal spends time in a herd with a history of bTB breakdowns in the previous 5 years, such that an animal that has spent some of its life in a herd with a recent history of bTB has twice the odds of being a SLH case than an animal that has spent no time in a herd with a recent history of bTB. This is likely to be because the history of bTB in a herd captures proxy risks for individual animals within those herds, such as being more likely to harbour hidden infections [9] or having localised reservoirs of infection. This is qualitatively similar to the earlier study in the ROI [10], where the risk of submission and confirmation (in general) was smaller in animals that were sourced from herds that had been clear of bTB infection for longer. In NI [12] the risk of disclosure seems to be smaller in herds that have recently cleared infection, but then rises again for herds that have been clear for longer. This may reflect differences in the underlying epidemiology, or effectiveness of testing, in NI compared to GB.

There is some marginal evidence of a seasonal effect. The lowest odds of SLH detection are found in April—June, with a slightly higher odds of SLH detection for animals slaughtered in October—December (OR of 1.2 on average), with a relatively smooth transition in the interim quarters. This seasonal variation in detection does not necessarily represent variability in the efficiencies of slaughterhouses. We think it is more likely to be driven by a changing pattern of presentation of lesioned animals as a consequence of seasonality in patterns of slaughter and testing, and note that the pattern is similar in ROI [10] but weaker in NI [12].

Interestingly, the more times that animals have been present on herds when the herd has been tested slightly increases the odds of subsequent detection as a slaughterhouse case, but the effect is not strong (OR of 1.2 on average, relative to an animals that have never been present for a test). Animals that remain on the same herd for the whole of their lives have a slightly higher odds of SLH detection (OR of 1.2 on average, relative to animals that have moved herds at least once). This contrasts with NI [12] where homebred animals (e.g. no movements) were at slightly lower risk of disclosure, but aligns with ROI [10] where the reverse was true. We note in all cases that the ORs were not strong, and in our case this pattern may be a consequence of the introduction of pre- and post-movement testing towards the end of our study period (2007 onwards). Finally, sex seems to play a negligible role, with female animals having slightly higher odds of being a SLH case than males (with an OR of 1.1 on average, but we note that the 95% credible interval contains 1). This is in line with ROI [10] (which found a negligible effect), and NI [12] (which found a stronger positive effect for females).

Over-and-above these risk factors there seems to be an effect of breed (Fig C S1 File). However these breed effects are much smaller than the corresponding SLH effects (see below), and were effectively treated as nuisance variables in these analyses.

#### SLH variability

**Fig 4:**
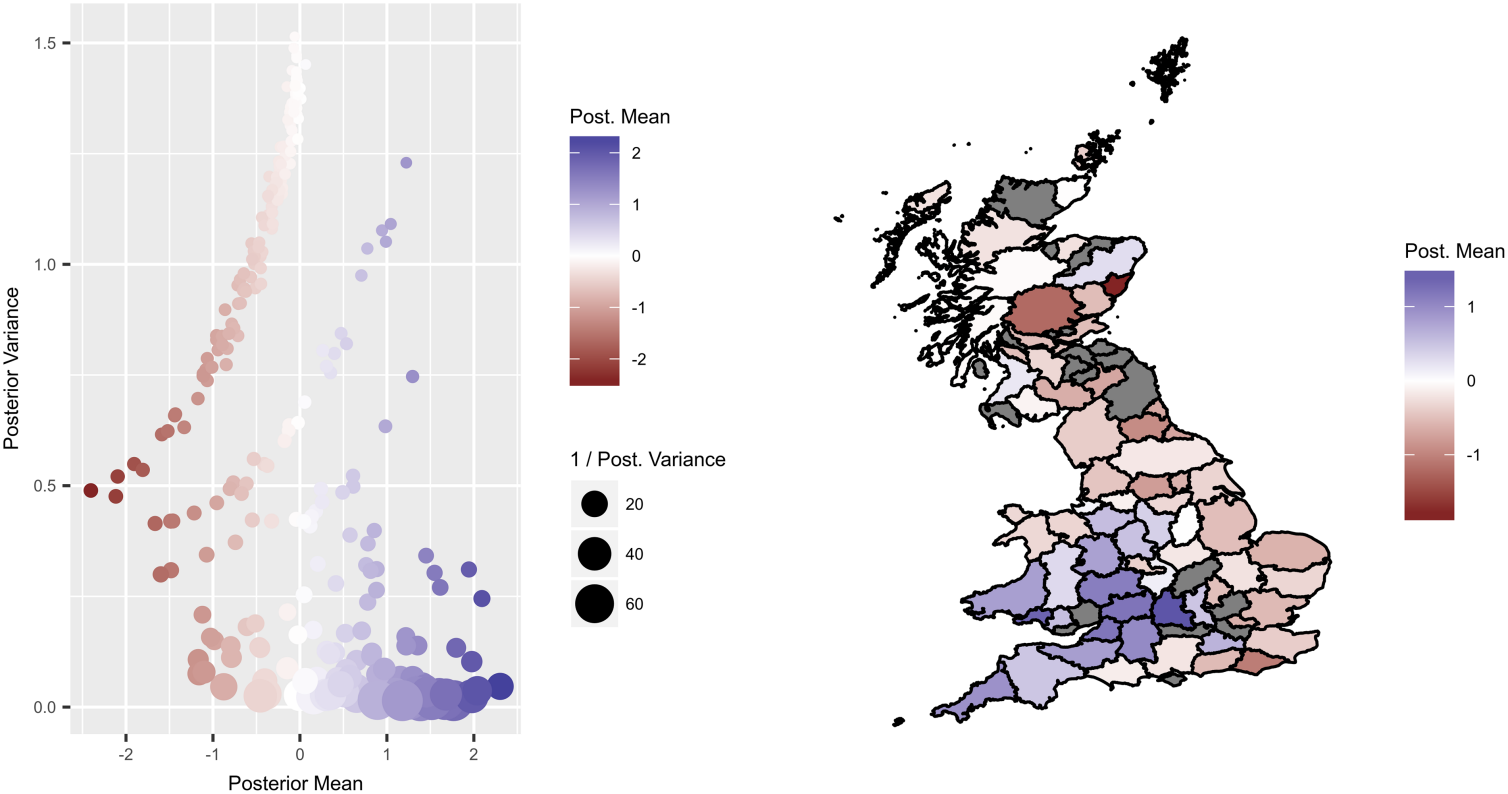
Slaughterhouse effects. Posterior variances against posterior means for the slaughterhouse hierarchical intercepts terms, shown against each other (left-hand panel) and mapped to the spatial locations of the slaughterhouses (right-hand panel). The larger the point, the more precisely the term has been estimated, with blue points corresponding to slaughterhouses that find more cases than expected, and red points corresponding to slaughterhouses that find less cases than expected, according to the fitted model.

Due to the large number of terms, the caterpillar plots for the SLH effects (Fig B S1 File) are hard to interpret. A key point is that the relative importance of each SLH is going to be related to both the posterior mean (how much each SLH deviates from the overall mean), and the posterior variance (how much uncertainty exists), and so in addition we produce a scatterplot of the posterior variance against the posterior mean for each SLH term (Fig 4). To aid interpretation, the point size is inversely related to the variance (so larger points have smaller variances), and the colours are mapped to the mean, such that blue points show an increased odds of picking up SLH cases, and the red points show a decreased odds (relative to the overall mean). To protect confidentiality, the spatial plot is aggregated at the county level.

The key SLHs of interest are those with large (absolute) means and small variances (i.e. those that deviate away from the mean with low uncertainty). It is important to remember that these values relate to deviations away from the overall mean as defined by the regression model, and we cannot assign a causal reason for these patterns. Rather, the model can be used to identify ‘atypical’ patterns across the slaughterhouses, which could be subjected to further investigation. The variables used in the regression were chosen to try to reflect different sources of risk for individual animals over their lifetime, but there is clearly some residual risk due to variables that we have not measured, which is captured in these SLH terms. To assess how much of the overall risk can be attributed to these unobserved effects, we refitted the model and excluded the SLH terms. This resulted in a posterior predictive mean estimate for the AUC of 0.85 compared to the posterior mean AUC rose of 0.88 for the full model. This suggests that the main explanatory variables chosen for the model do a good job of capturing most of the risk, but that there is some additional heterogeneity between slaughterhouses.

One possibility is that these SLH effects could result from variability in the underlying disease prevalence of animals sent to particular slaughterhouses. For example, those slaughterhouses sourcing animals from high-risk areas are more likely to find ‘hidden’ infections than those sourcing from low-risk areas. Plotting these estimates according to the point location of each slaughterhouse suggests that there is some spatial correlation, such that on average slaughterhouses situated in high-risk areas (south-west England and Wales) find more SLH cases than those situated in low-risk areas (Fig 4), despite those areas testing more frequently. (We note that the spatial location of a slaughterhouse is not necessarily indicative of where the animals are sourced from. It is possible to send animals to slaughterhouses that are located some distance from the farm, but generally farmers tend to send animals to SLHs that are situated locally). Another possibility is that there are differences in the detection rates within slaughterhouses due to factors such as training or throughputs. Of particular interest are slaughterhouses that have a lower-than-average detection rate situated in high-risk areas, and those that have a higher-than-average detection rate situated in low-risk areas.

An important interpretational consideration is that the form of the model itself defines the structure of these SLH terms, such that we would always expect deviations around the true mean. (We model these terms using a hierarchical normal distribution with a shared variance term (on the logistic scale), so by definition we would expect spread around the overall mean). The key point is the magnitude of these differences. The posterior mean odds ratios range from between 0.11 to 10 relative to the overall mean, which represents an epidemiologically important level of variability between (a small number) of slaughterhouses. (If there were small differences we would still see a spread around the mean, but the range would be much smaller—as is seen in the breed effects, which range between 0.65 and 1.7 on the same scale).

## Conclusions

We describe an increase in both the absolute number and proportion of breakdowns disclosed through slaughterhouse surveillance in Great Britain between 1998 and 2013. This increase remains even when accounting for changes in confirmation rates and changes in the proportion of herds subject to different testing frequencies.

We used a case-control study to explore the impact of individual animal-level factors on the risk of an animal being identified as a slaughterhouse case. We showed that after adjusting for other sources of risk there are residual between-slaughterhouse variabilities in detection rates. There is also an additional increased risk over time of animals being detected as slaughterhouse cases that is not explained by the available risk factors. This variability is not necessarily due to differences in practice within individual slaughterhouses, but may manifest due to systematic differences in the risk of infection or efficiency of tuberculin testing within the input population for each slaughterhouse. As such, the key use for this model is to identify ‘atypical’ slaughterhouses (i.e. ones that exhibit in inflated or deflated detection rates relative to the overall mean predicted by the model). Hence, although our model cannot assign causal reasons for these differences, it will act as a guide for further investigations. Currently the Animal and Plant Health Agency have adopted the model to be used as part of their ongoing bTB surveillance programme.

## Supporting information

S1 File Supplementary information. Includes all code necessary to rerun analyses, plus additional plots and tables not included in the main text.
(PDF)

S1 Data A data file (.rds) containing the raw data. (RDS)

## References

[1] Defra. Freedom of information request; 2014. Available from: https://www.gov.uk/government/uploads/system/uploads/attachment_data/file/323911/RFI_6505.pdf.

[2] Abernethy DA, Upton P, Higgins IM, McGrath G, Goodchild AV, Rolfe SJ, et al Bovine tuberculosis trends in the UK and the Republic of Ireland, 1995–2010. Veterinary Record. 2013;172(12):312 doi: 10.1136/vr.100969 23292950 10.1136/vr.100969

[3] Broughan JM, Brouwer A, Upton P. Analysis of bovine tuberculosis surveillance at routine slaughter of cattle in Great Britain (2009–2013); 2014. Available from: https://www.gov.uk/government/uploads/system/uploads/attachment_data/file/376705/tb-pub-surveport-slauhou-09-13-19nov14.pdf.

[4] Defra. Testing regions; 2018. Available from: https://assets.publishing.service.gov.uk/government/uploads/system/uploads/attachment_data/file/662374/pti-map18.pdf.

[5] Goodchild AV, Downs SH, Upton P, Wood JLN, de la Rua-Domenech R. Specificity of the comparative skin test for bovine tuberculosis in Great Britain. The Veterinary Record. 2015;177(10):258 doi: 10.1136/vr.102961 26338518 10.1136/vr.102961 PMC4602248

[6] de la Rua-Domenech R, Goodchild AT, Vordermeier HM, Hewinson RG, Christiansen KH, Clifton-Hadley RS. Ante mortem diagnosis of tuberculosis in cattle: A review of the tuberculin tests, *γ*-interferon assay and other ancillary diagnostic techniques. Research in Veterinary Science. 2006;81(2):190–210. doi: 10.1016/j.rvsc.2005.11.005 16513150 10.1016/j.rvsc.2005.11.005

[7] Karolemeas K, de la Rua-Domenech R, Cooper R, Goodchild AV, Clifton-Hadley RS, Conlan AJK, et al Estimation of the Relative Sensitivity of the Comparative Tuberculin Skin Test in Tuberculous Cattle Herds Subjected to Depopulation. PLoS ONE. 2012;7(8):e43217 doi: 10.1371/journal.pone.0043217 22927952 10.1371/journal.pone.0043217 PMC3424237

[8] Mitchell AP, Green LE, Clifton-Hadley R, Mawdsley J, Sayers R, Medley GF. Analysis of single intradermal comparative cervical test (SICCT) coverage in the GB cattle population. Proceedings Society of Veterinary Epidemiology and Preventive Medicine. 2006; p. 70–86.

[9] Conlan AJ, McKinley TJ, Karolemeas K, Pollock EB, Goodchild AV, Mitchell AP, et al Estimating the hidden burden of bovine tuberculosis in Great Britain. PLoS Comput Biol. 2012;8(10):e1002730 doi: 10.1371/journal.pcbi.1002730 23093923 10.1371/journal.pcbi.1002730 PMC3475695

[10] Frankena K, White PW, O’Keeffe J, Costello E, Martin SW, van Grevenhof I, et al Quantification of the relative efficiency of factory surveillance in the disclosure of tuberculosis lesions in attested Irish cattle. Veterinary Record. 2007;161(20):679–684. doi: 10.1136/vr.161.20.679 18024922 10.1136/vr.161.20.679

[11] Olea-Popelka F, Freeman Z, White P, Costello E, O’Keeffe J, Frankena K, et al Relative effectiveness of Irish factories in the surveillance of slaughtered cattle for visible lesions of tuberculosis, 2005–2007. Irish Veterinary Journal. 2012;65:2 doi: 10.1186/2046-0481-65-2 22289139 10.1186/2046-0481-65-2 PMC3311595

[12] Pascual-Linaza AV, Gordon AW, Stringer LA, Menzies FD. Efficiency of slaughterhouse surveillance for the detection of bovine tuberculosis in cattle in Northern Ireland. Epidemiology and Infection. 2012;145:995–1005. doi: 10.1017/S0950268816003095 10.1017/S0950268816003095 PMC9507821 28027717

[13] Shittu A, Clifton-Hadley RS, Ely ER, Upton PU, Downs SH. Factors associated with bovine tuberculosis confirmation rates in suspect lesions found in cattle at routine slaughter in Great Britain, 2003–2008. Preventive Veterinary Medicine. 2013;110(3–4):395–404. doi: 10.1016/j.prevetmed.2013.03.001 23540447 10.1016/j.prevetmed.2013.03.001

[14] Allen AR, Minozzi G, Glass EJ, Skuce RA, McDowell SWJ, Woolliams JA, et al Bovine tuberculosis: the genetic basis of host susceptibility. Proceedings of the Royal Society of London B: Biological Sciences. 2010; p. 2737–2745. doi: 10.1098/rspb.2010.0830 10.1098/rspb.2010.0830 PMC2981996 20519223

[15] Downs SH, Broughan JM, Goodchild AV, Upton PA, Durr PA. Responses to diagnostic tests for bovine tuberculosis in dairy and non-dairy cattle naturally exposed to Mycobacterium bovis in Great Britain. The Veterinary Journal. 2016;216:8–17. doi: 10.1016/j.tvjl.2016.06.010 27687920 10.1016/j.tvjl.2016.06.010

[16] R Core Team. R: A Language and Environment for Statistical Computing; 2016. Available from: https://www.R-project.org/.

[17] Karolemeas K, McKinley TJ, Clifton-Hadley RS, Goodchild AV, Mitchell A, Johnston WT, et al Recurrence of bovine tuberculosis breakdowns in Great Britain: risk factors and prediction. Preventive Veterinary Medicine. 2010; p. 22–29. 10.1016/j.prevetmed.2011.06.004 21767886

[18] Skuce RA, Allen AR, McDowell SWJ. Herd-level risk factors for bovine tuberculosis: a literature review. Veterinary Medicine International. 2012;2012 doi: 10.1155/2012/621210 22966479 10.1155/2012/621210 PMC3395266

[19] Gelman A, Hill J, Yajima M. Why We (Usually) Don’t Have to Worry About Multiple Comparisons. Journal of Research on Educational Effectiveness. 2012;5(2):189–211. doi: 10.1080/19345747.2011.618213

[20] Papaspiliopoulous O, Roberts GO, Sköld M. Non-centered parameterizations for hierarchical models and data augmentation In: Bernardo JM, Bayarri MJ, Berger JO, Dawid AP, Heckerman D, Smith AFM, et al, editors. Bayesian Statistics 7. Oxford University Press; 2003 p. 307–326.

[21] Gelman A, Hill J. Data Analysis Using Regression and Multilevel/Hierarchical Models. Cambridge University Press; 2006.

[22] Hosmer DW, Lemeshow S. Applied Logistic Regression. John Wiley & Sons; 2004.

