# Supplementary material for: Risk factors and variations in detection of new bovine tuberculosis breakdowns via slaughterhouse surveillance in Great Britain
Source: PLoS One. 2018 Jun 8;13(6):e0198760. doi: 10.1371/journal.pone.0198760 (PMC5993271; doi:10.1371/journal.pone.0198760)
Supplement: S1 File — Includes all code necessary to rerun analyses, plus additional plots and tables not included in the main text. (PDF) [file pone.0198760.s001.pdf]

Supplementary Materials: Risk factors associated with the detection of new TB breakdowns via abattoir surveillance in Great Britain

Trevelyan J. McKinley, Debby Lipschutz-Powell, Andrew P. Mitchell, James L. N. Wood and Andrew J. K. Conlan

This is a working document to try to perform analyses for our paper, and to aid reproducibility. All of the code has been packaged into an R package called `BayesLog`.

Acknowledgements

All the models were developed and run using RStudio (RStudio Team 2015) and the R statistical language (R Core Team 2016). This document was written using the `rmarkdown` (Allaire et al. 2016) and `knitr` (Xie 2015) packages, with useful features provided by `kfigr` (Koohafkan 2015) and `pander` (Daróczi and Tsegelskyi 2015).

In addition to the dependencies listed in the `BayesLog` package: `Rcpp` (Eddelbuettel 2013; Eddelbuettel and Francois 2011), `RcppArmadillo` (Eddelbuettel and Sanderson 2014), `coda` (Plummer et al. 2006), `lme4` (Bates et al. 2015), `caTools` (Tuszynski 2014), `ggplot2` (Wickham 2009), `dplyr` (Wickham and Francois 2016), `circlize` (Z. Gu et al. 2014), `grid` (R Core Team 2016), `stats` (R Core Team 2016) and `gridExtra` (Auguie 2017), we also used `arm` (Gelman and Su 2015), `maptools` (Bivand and Lewin-Koh 2016), `reshape2` (Wickham 2007) and `scales` (Wickham 2016) to produce various outputs/figures in this document.

Contact days

Figure A shows the distributions of ln(contact days+1) for animals in low-risk and high-risk herds respectively. Due to the strong bimodality, we categorised these into "None" or "Some" presence when creating the data.

Analysis

Firstly, load the necessary libraries:

```
library(BayesLog)
library(arm)
library(ggplot2)
library(reshape2)
library(maptools)
library(grid)
library(scales)
library(dplyr)
```

Regression model

These data have been collated into a dataframe called `df_ad`. Secondly, we load in the data:

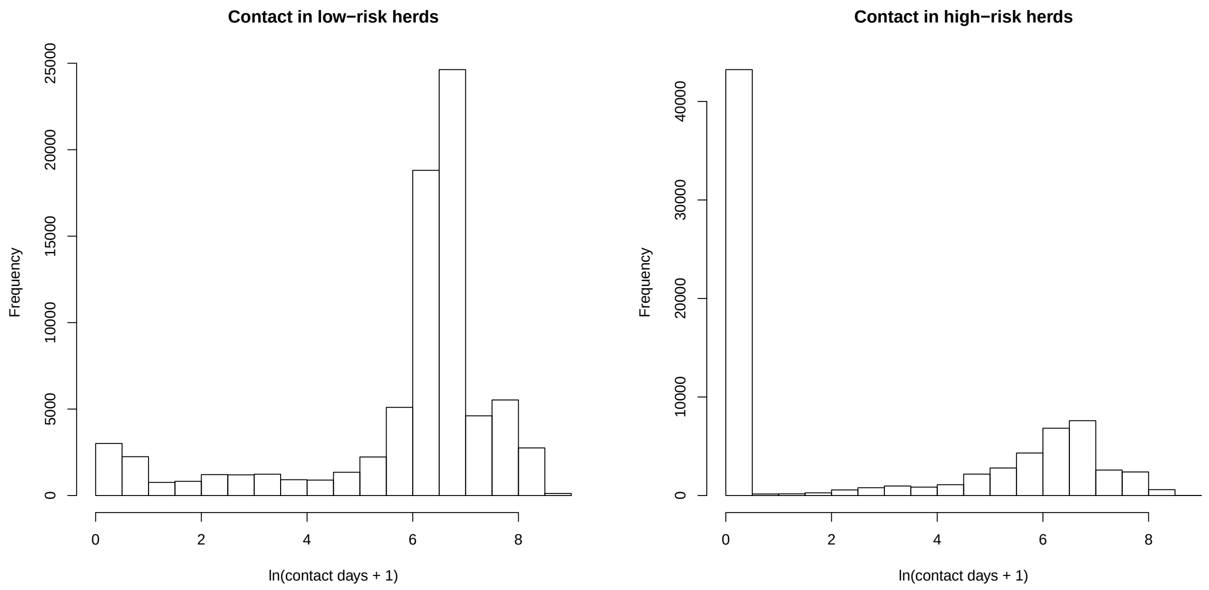

Figure A: Histograms of ln(contact days + 1) for animals in low-risk and high-risk herds respectively.

```
## load data and summarise
df_ad <- readRDS("S1 Data.rds")
summary(df_ad)
```

```
      SLH            Breed           SLHcase         Sex
 164     : 3149   LIMX    :16428   Min.   :0.0000   M:38565
 240     : 3056   HF      :11364   1st Qu.:0.0000   F:38861
 242     : 2957   CHX     : 9012   Median :0.0000
 231     : 2849   SMX     : 5445   Mean   :0.1112
 4       : 2632   AAX     : 5097   3rd Qu.:0.0000
 134     : 2290   BBX     : 3557   Max.   :1.0000
 (Other):60493   (Other):26523
      Year            Quarter            RiskReg         MoveInd
 Min.   : 1.000   Apr-Jun:18376   L_Scot  :15856   >=1:58228
 1st Qu.: 3.000   Jul-Sep:18640   L_Eng   :26103   0  :19198
 Median : 6.000   Oct-Dec:21253   L_Wales: 3827
 Mean   : 5.575   Jan-Mar:19157   H_Wales: 6464
 3rd Qu.: 8.000                   H_Eng   :25176
 Max.   :10.000

      Age          Contact_Low  Contact_High TestPresent
 (0,18]  :12504   None: 3013   None:43228   0 :23177
 (18,24]:17872   Some:74413   Some:34198   1 :11847
 (24,36]:28921                              1+:42402
 (36,60]: 4470
 >60     :13659
```

The `df_ad` dataset consists of 77426 observations: 8607 cases and 68819 controls. The cases correspond to *all* animals that were slaughterhouse cases between the years 2004–2013, obtained from 276 slaughterhouses.

The controls were obtained using stratified sampling, such that 0.3% of the total control population in each of the 276 slaughterhouses was sampled. Summaries of the number of samples stratified by `SLH` and `Breed` effects are shown in Tables A and B respectively. (Note that these have been ordered in terms of highest number of samples to lowest, and the SLHs and breeds comprising the lowest 25–30% of the samples have been grouped together to save space. All slaughterhouse IDs have been anonymised by randomly assigning the numbers 1–276 between the slaughterhouses.)

Table A: Summaries of number of samples by slaughterhouse

SLH indicator	Number of samples	Cumulative proportion
164	3149	0.041
240	3056	0.08
242	2957	0.12
231	2849	0.16
4	2632	0.19
134	2290	0.22
39	2245	0.25
54	2201	0.28
67	2096	0.3
251	1922	0.33
107	1844	0.35
2	1778	0.37
28	1761	0.4
208	1594	0.42
174	1568	0.44
230	1462	0.46
108	1449	0.48
252	1403	0.49
232	1373	0.51
76	1301	0.53
207	1243	0.54
124	1219	0.56
154	1190	0.58
63	1171	0.59
160	1122	0.61
201	1119	0.62
255	967	0.63
33	948	0.64
157	888	0.66
62	825	0.67
128	812	0.68
121	767	0.69
184	699	0.7
268	666	0.7
101	650	0.71
156	603	0.72
1	577	0.73
254	548	0.74
200	530	0.74
Others	19422	1

Table B: Summaries of number of samples by breed

Breed	Number of samples	Cumulative proportion
LIMX	16428	0.21
HF	11364	0.36
CHX	9012	0.48
SMX	5445	0.55
AAX	5097	0.61
BBX	3557	0.66
BF	3151	0.7
Others	21011	1

The variables in the final data set are described in Table C.

Table C: Animal-level explanatory variables in model

Sex	Sex of animal	Categorical	M / F
Year	Year post 2003 that animal was slaughtered	Integer	1-10
Quarter	Quarter that animal was slaughtered	Categorical	Apr–Jun, . . . , Jan–Mar
RiskReg	Region animal was present for most of its life	Categorical	LFT in Scotland / England / Wales HFT in Wales / England
MoveInd	No. of movements between herds	Categorical	1+, 0
Age	Age at slaughter (months)	Categorical	0-18, 18-24, 24-36, 36-60, >60
Contact_Low	Contact days in low risk herd	Categorical	None / Some
Contact_High	Contact days in high risk herd	Categorical	None / Some
TestPresent	Present for herd test	Categorical	0, 1, 1+
SLH	Slaughterhouse indicator (where the animal was slaughtered)	Categorical	276 levels
Breed	Breed	Categorical	166 levels
SLHcase	SLH case	Binary	0 / 1 (no / yes)

Bayesian hierarchical model

The response variable is:

$$Y_i = \begin{cases} 1 & \text{if animal } i \text{ is SLH case,} \\ 0 & \text{otherwise.} \end{cases}$$

Hence $Y_i \sim \text{Bin}(p_i)$ where

$$\log\left(\frac{p_i}{1-p_i}\right) = \beta^T \mathbf{x}_i + \theta_{B_i} + \gamma_{S_i},$$

where the $\mathbf{x}_i$ are a vector of explanatory variables shown in Table C, and B_i and S_i correspond to the breed and slaughterhouse for individual i. The regression parameters are given by β, and θ_k and γ_l are the hierarchical terms (analogous to a random intercepts model). In this case we set the prior distributions as:

$$\begin{aligned}
\beta_j &\sim N\left(0, 100^2\right), \\
\theta_k &\sim N\left(0, \sigma_\theta^2\right), \\
\gamma_l &\sim N\left(0, \sigma_\gamma^2\right), \\
\sigma_\theta &\sim U(0, 20), \\
\sigma_\gamma &\sim U(0, 20).
\end{aligned}$$

To fit this model, we have written a function `bayesLog()`, that runs the model and produces a `bayesLog` object, for which we have implemented various generic methods, including: `plot`, `summary`, `print`, `window` and `subset`. Please see the help files for information on each of these methods (e.g. `?bayesLog`). To specify the form of the model, we can use an R `formula` object, familiar to users of `lme4` and particularly `glmer()`. (Note that we can only implement hierarchical *intercepts* here, using the syntax `(1 | variable)`.)

To generate a formula for this model we use:

```
## generate formula for GLM
predictors <- colnames(df_ad)
predictors <- predictors[-match(c("SLH", "Breed", "SLHcase"),
predictors)]

## update formula to include hierarchical intercepts
model.form <- formula(paste("SLHcase ~ 1 +",
                paste(predictors, collapse = " + "), "+ (1 | Breed) + (1 | SLH)"))
model.form
```

```
SLHcase ~ 1 + Sex + Year + Quarter + RiskReg + MoveInd + Age +
    Contact_Low + Contact_High + TestPresent + (1 | Breed) +
    (1 | SLH)
```

To run the model and discard the burn-in we use:

```
## run Bayesian model
model.mcmc <- bayesLog(model.form, df_ad, priorvar = 10000,
niter = 10000, nprintsum = 5000, noncentreint = "all")

## discard burn-in
model.mcmc <- window(model.mcmc, start = 5000)
```

```
Number of samples in data set = 77426
Number of unique samples in data set = 69558

Run time information printed to screen every 5000 iterations
Number of iterations = 10000
Scale for adaptive proposal = 0.05
Number of regression parameters = 21
Number of random intercepts = 2
Adapt every 100 iterations
Max scale for adapting = 2.00
1000 iterations before diminishing adaptation kicks in

Priors: mean = 0.00 variance = 10000.00
Priors RE SD: lower = 0.00 upper = 20.00
```

```
Starting run:
i = 5000 minacc = 0.41 maxacc = 0.45 minaccnon = 0.24 maxaccnon = 0.29
    minacc_rand = 0.44 maxacc_rand = 0.50 minacc_randnon = 0.25 maxacc_randnon = 0.28
    ime = 4384.51
i = 10000 minacc = 0.44 maxacc = 0.45 minaccnon = 0.25 maxaccnon = 0.26
    minacc_rand = 0.44 maxacc_rand = 0.45 minacc_randnon = 0.25 maxacc_randnon = 0.26
    ime = 373.92
Total run time = 4758.43

Starting run:
i = 5000 minacc = 0.41 maxacc = 0.45 minaccnon = 0.24 maxaccnon = 0.28
    minacc_rand = 0.44 maxacc_rand = 0.50 minacc_randnon = 0.25 maxacc_randnon = 0.28
    ime = 436.55
i = 10000 minacc = 0.44 maxacc = 0.45 minaccnon = 0.25 maxaccnon = 0.26
    minacc_rand = 0.44 maxacc_rand = 0.45 minacc_randnon = 0.25 maxacc_randnon = 0.26
    ime = 431.59
Total run time = 868.14
```

(Note that we found the mixing and convergence improved if we used a combination of centred and non-centred updates, specified here by the use of `noncentreint = "all"`—see e.g. Papaspiliopoulos, Roberts, and Sköld (2003)—full details are given in the Appendix to this document.)

We can produce trace plots for the coefficients as:

```r
## plot trace plots for just the regression parameters
plot(model.mcmc, density = F)

## trace plots for the hierarchical intercepts
plot(model.mcmc[, "SLH"])
plot(model.mcmc[, "Breed"])
```

For brevity we show the trace plots for the regression terms in the Appendix, but do not show the `SLH` or `Breed` effects here. In addition, we check the overall model fit using a binned residual plot (Gelman and Hill 2007) (using the posterior predictive means from the fitted model as point estimates—see Appendix). The plots good convergence and mixing, and there is no strong systematic pattern to the residuals compared to the predictions from the model.

Results

Once we are happy with the convergence, we can summarise the results as follows:

```r
summary(model.mcmc)
```

```
Iterations = 5000:10000
Thinning interval = 1
Number of chains = 2
Sample size per chain = 5001

1. Empirical mean and standard deviation for each variable,
   plus standard error of the mean:

                     Mean       SD  Naive SE Time-series SE
(Intercept)      -7.82827 0.223812 2.238e-03      0.0079421
```

```
SexF              0.05195 0.032616 3.261e-04      0.0010141
Year              0.10303 0.005633 5.632e-05      0.0002830
QuarterJul-Sep    0.06476 0.038827 3.882e-04      0.0009160
QuarterOct-Dec    0.18234 0.036931 3.693e-04      0.0008624
QuarterJan-Mar    0.06761 0.038375 3.837e-04      0.0008892
RiskRegL_Eng      1.86313 0.182401 1.824e-03      0.0067190
RiskRegL_Wales    1.95989 0.193623 1.936e-03      0.0075697
RiskRegH_Wales    2.49844 0.186209 1.862e-03      0.0070354
RiskRegH_Eng      3.02797 0.181207 1.812e-03      0.0072355
MoveInd0          0.15178 0.030718 3.072e-04      0.0007474
Age(18,24]        0.67602 0.067137 6.713e-04      0.0017581
Age(24,36]        0.89327 0.063046 6.304e-04      0.0017767
Age(36,60]        1.22718 0.080488 8.048e-04      0.0022874
Age>60            1.66336 0.075384 7.538e-04      0.0026014
Contact_LowSome   0.08081 0.055622 5.562e-04      0.0014734
Contact_HighSome  0.70898 0.037002 3.700e-04      0.0010693
TestPresent1      0.01239 0.072219 7.221e-04      0.0015591
TestPresent1+     0.18290 0.052535 5.253e-04      0.0014856
Breedsd           0.29242 0.039041 3.904e-04      0.0021554
SLHsd             1.19740 0.089198 8.919e-04      0.0044010

2. Quantiles for each variable:

                      2.5%      25%      50%      75%   97.5%
(Intercept)      -8.280744 -7.97513 -7.82137 -7.67681 -7.4011
SexF             -0.014897  0.03105  0.05224  0.07355  0.1161
Year              0.092213  0.09922  0.10302  0.10684  0.1139
QuarterJul-Sep   -0.010410  0.03934  0.06508  0.09031  0.1415
QuarterOct-Dec    0.109929  0.15695  0.18279  0.20729  0.2549
QuarterJan-Mar   -0.005523  0.04157  0.06739  0.09274  0.1441
RiskRegL_Eng      1.507839  1.74514  1.86172  1.98424  2.2144
RiskRegL_Wales    1.583042  1.82965  1.95971  2.09118  2.3472
RiskRegH_Wales    2.133686  2.37984  2.49852  2.62344  2.8602
RiskRegH_Eng      2.683769  2.90888  3.02672  3.14969  3.3780
MoveInd0          0.093814  0.13116  0.15178  0.17260  0.2135
Age(18,24]        0.545023  0.63041  0.67545  0.72064  0.8060
Age(24,36]        0.770773  0.85055  0.89228  0.93590  1.0177
Age(36,60]        1.067943  1.17186  1.22686  1.28086  1.3855
Age>60            1.518298  1.61205  1.66286  1.71556  1.8093
Contact_LowSome  -0.025297  0.04127  0.07917  0.11882  0.1907
Contact_HighSome  0.636707  0.68330  0.70926  0.73392  0.7812
TestPresent1     -0.127329 -0.03721  0.01325  0.06091  0.1542
TestPresent1+     0.079130  0.14770  0.18146  0.21770  0.2914
Breedsd           0.225691  0.26429  0.28893  0.31780  0.3767
SLHsd             1.040082  1.13414  1.19262  1.25518  1.3859
```

We can also produce caterpillar plots for the hierarchical terms (Figures B and C):

```
## function to produce plots of hierarchical intercepts posteriors
catplot <- function(model.mcmc, randnames, sideways = F)
{
  ## plot posteriors for random intercepts
  y <- as.matrix(model.mcmc[, randnames])

  ## turn into data frame to use in ggplot
```

```
  y <- as.data.frame(y)
  y <- melt(y)
  colnames(y) <- c("var", "nu")

  ## reset names
  y$var <- sub(randnames, "", as.character(y$var))
  y$var <- as.factor(y$var)

  ## sort according to posterior mean
  y$var <- with(y, reorder(var, nu, mean))

  ## export ggplot object
  p <- ggplot(y, aes(var, nu)) + geom_boxplot() + xlab(randnames) +
      ylab("Posterior Mean Log-Odds Ratios") +
      geom_hline(yintercept = 0, colour = "red", linetype = "dashed", size = 1.5)
  if(sideways) {
      p <- p + coord_flip()
  }
  p
}
```

```
## draw plots
print(catplot(model.mcmc, "SLH", T))
```

```
## draw plots
print(catplot(model.mcmc, "Breed", T))
```

We can see that these are hard to visualise, since coefficients with large posterior variances are likely to reflect lower throughputs, and so classifying slaughterhouses based solely on their posterior means is perhaps not sensible. To this end we also produce a plot of the posterior variances against the posterior means for each slaughterhouse, and also map these according to the spatial location of the slaughterhouse (Figure D). (Although slaughterhouses can source animals from anywhere across the country, the majority of animals are sourced from farms that are in close spatial proximity to the abattoir.) Note that for confidentiality reasons we do not include the code or the data for reproducing the spatial plots here.

To produce predictions we have implemented a generic `predict` function for `bayesLog` objects:

```
## generate predictive posterior from 1000 samples
## (500 from each chain)
inds <- nrow(as.matrix(model.mcmc)) / 2
inds <- sample(1:inds, 500)
model.pred <- predict(model.mcmc[inds, ], model.mcmc$data)
rm(inds)
```

From this we can generate a posterior predictive distribution for the AUC:

```
model.auc <- AUC(model.pred, model.mcmc$data$SLHcase)
model.auc
```

```
   Min. 1st Qu.  Median    Mean 3rd Qu.    Max.
 0.8797  0.8802  0.8804  0.8803  0.8805  0.8810
```

and can also calculate the sensitivities, specificities, positive predictive values and negative predictive values for a range of probability thresholds—see Figure E.

```
model.class <- classify(model.pred, model.mcmc$data$SLHcase, seq(0, 1, by = 0.05))
plot(model.class, "comp")
```

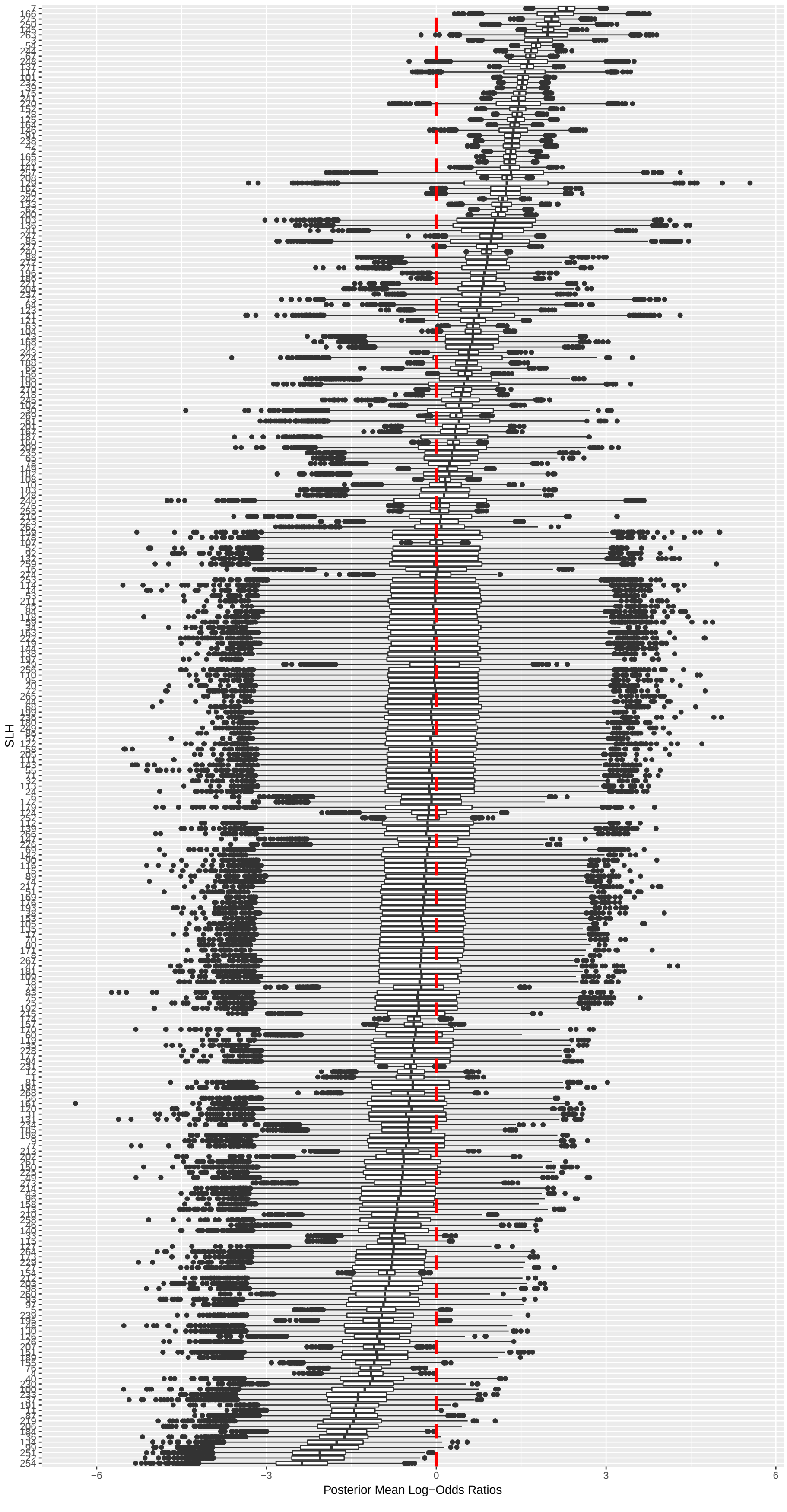

Figure B: Posterior distributions for the SLH hierarchical intercepts (ordered by posterior means)

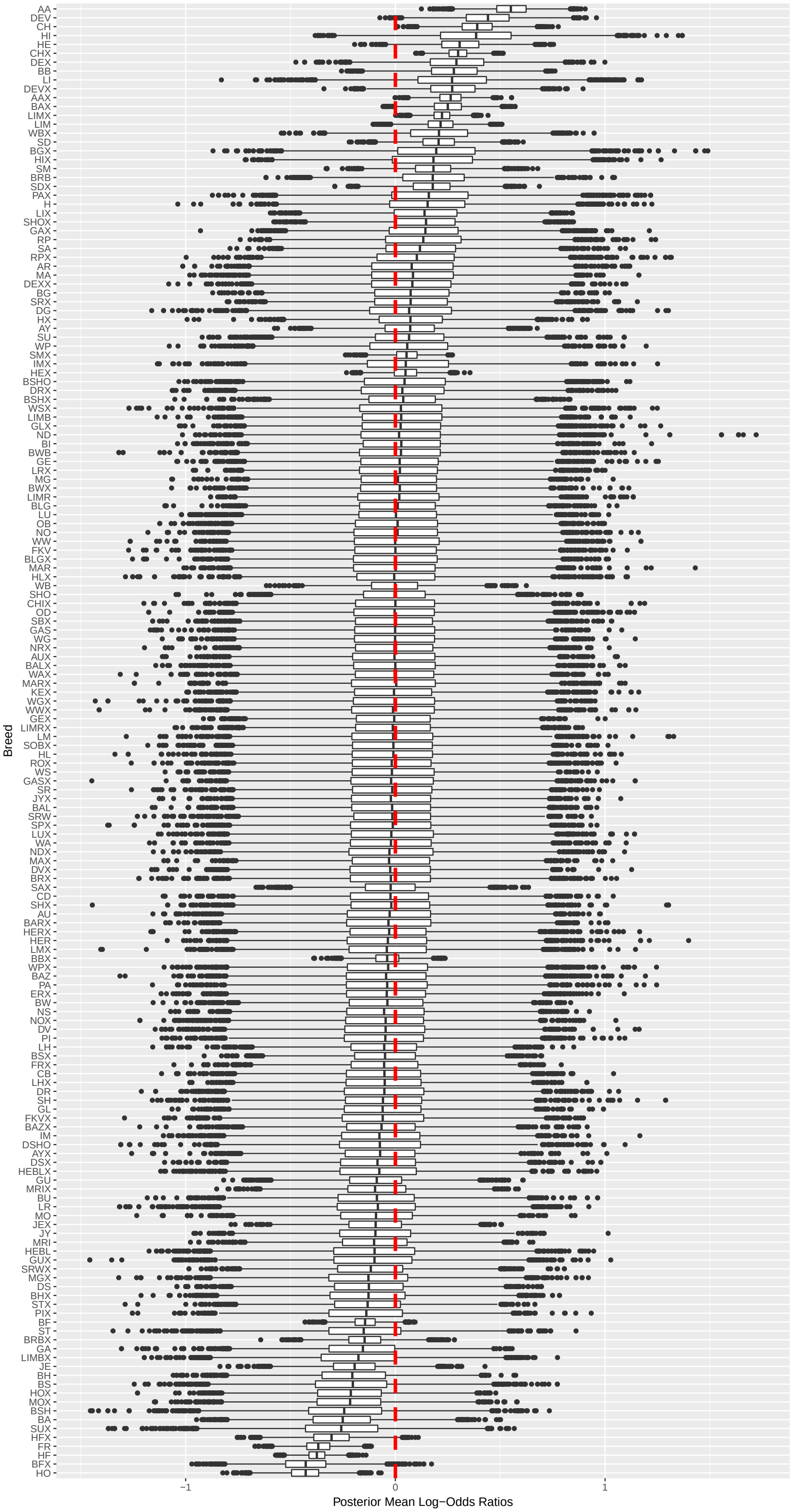

Figure C: Posterior distributions for the breed hierarchical intercepts (ordered by posterior means)

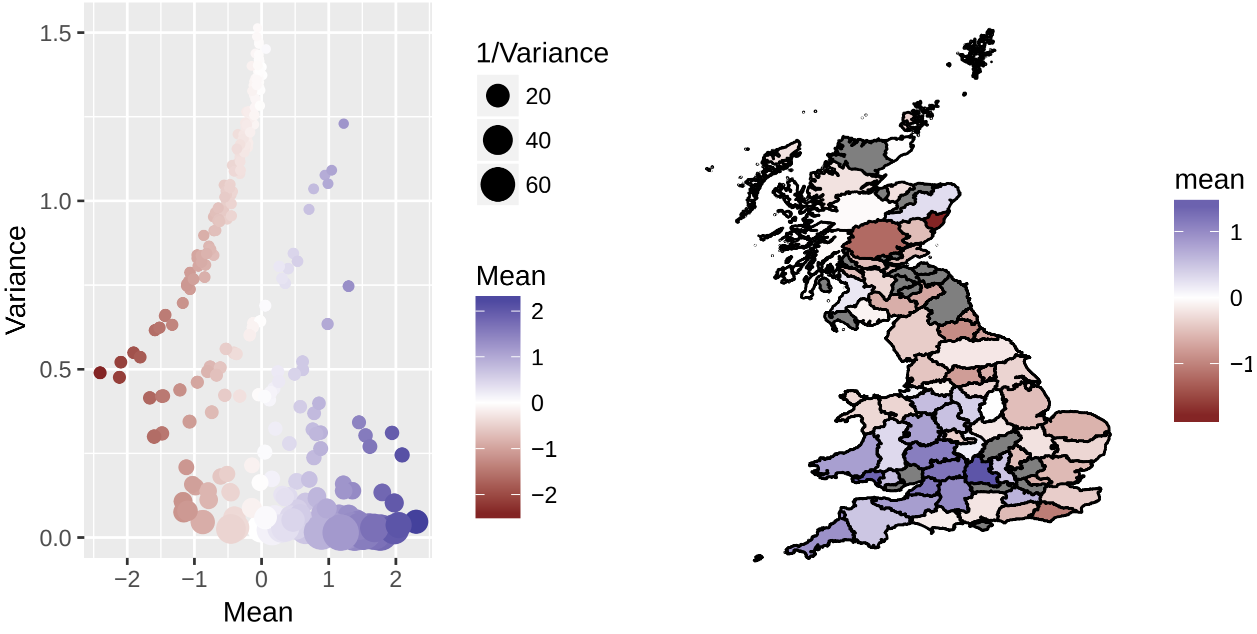

Figure D: Slaughterhouse intercept terms

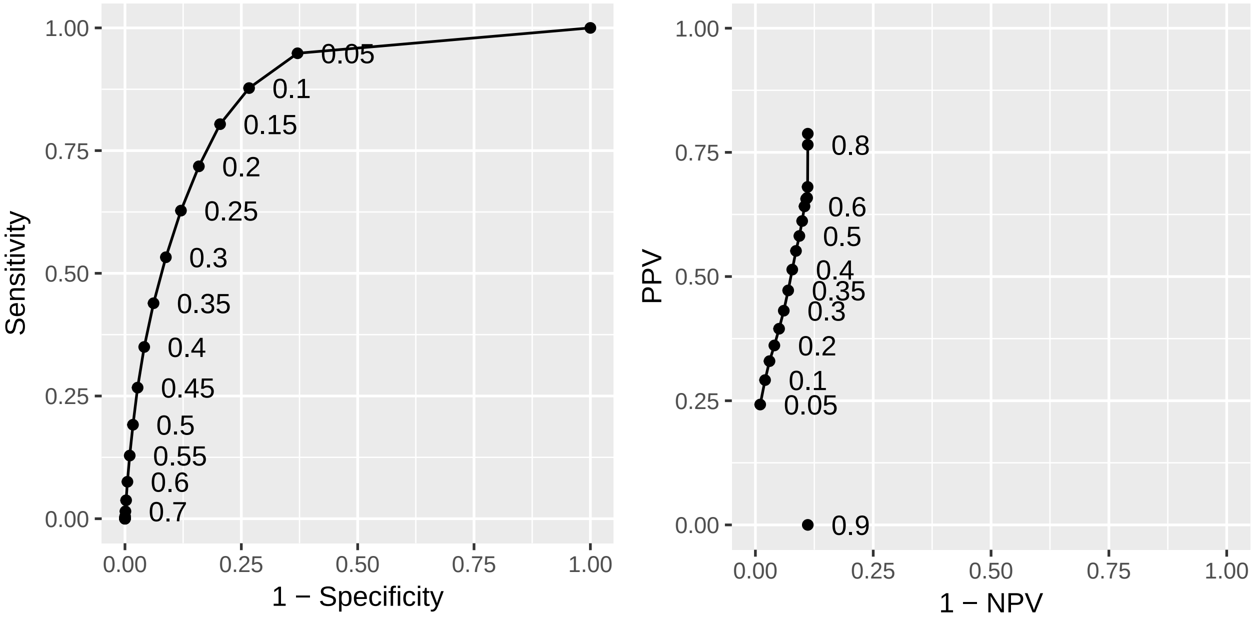

Figure E: Classification plots

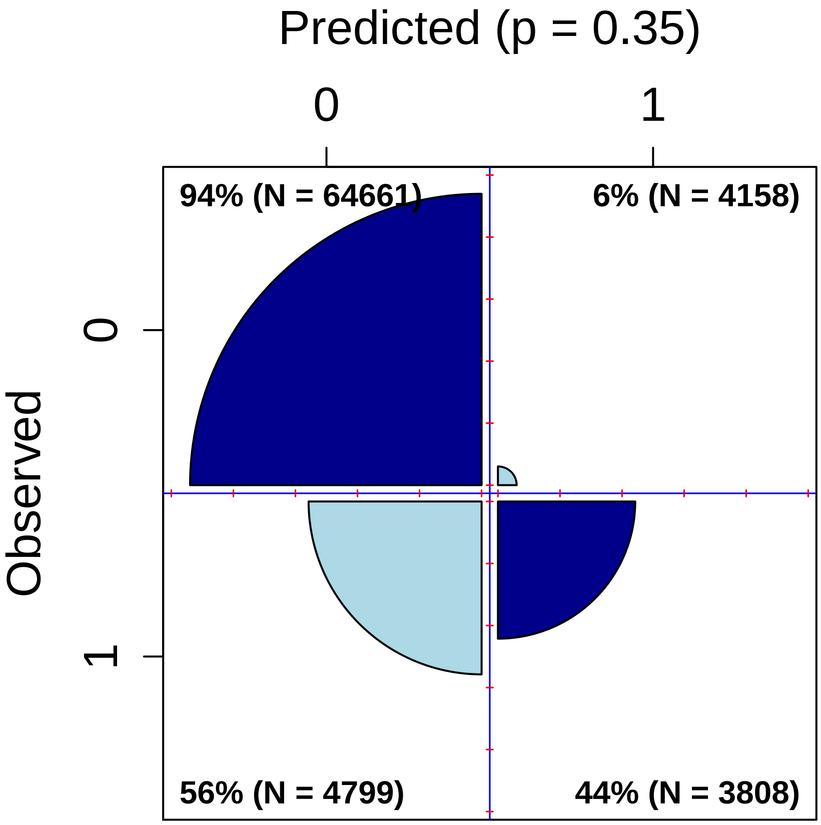

Figure F: Confusion matrix for threshold of 0.35

For a given threshold, we can also generate a confusion matrix (which summarises the overall misclassification rate of the model predictions—see Figure F).

```
confusion(model.pred, model.mcmc$data$SLHcase, 0.35)
```

```
   Pred
Obs      0      1
  0 64661   4158
  1   4799   3808
```

Comparison to model without SLH effects

It is also worth exploring how much of the predictive power is being driven by the SLH effects. To this end we can also refit the model after dropping these terms. (Note that we remove some of the output for brevity. Trace plots and binned residual plots are shown in the Appendix.)

```
## generate formula for GLM
model0.form <- formula(paste("SLHcase ~ 1 +",
                paste(predictors, collapse = " + "), "+ (1 | Breed)"))

## run Bayesian model
model0.mcmc <- bayesLog(model0.form, df_ad, priorvar = 10000,
niter = 10000, nprintsum = 5000, noncentreint = "all")

## discard burn-in
```

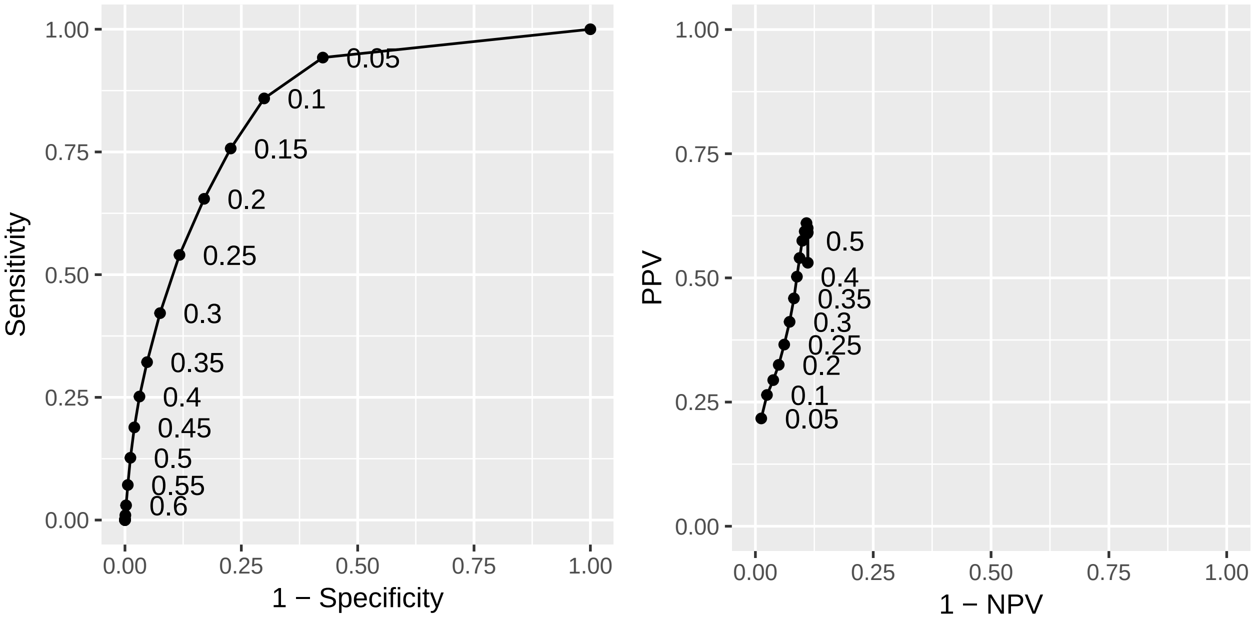

Figure G: Classification plots

```
model0.mcmc <- window(model0.mcmc, start = 5000)
```

Predictions and classification statistics are shown below and in Figure G:

```
## generate predictive posterior from 2000 samples
## (1000 from each chain)
inds <- nrow(as.matrix(model0.mcmc)) / 2
inds <- sample(1:inds, 500)
model0.pred <- predict(model0.mcmc[inds, ], model0.mcmc$data)
rm(inds)

## generate AUC
model0.auc <- AUC(model0.pred, model0.mcmc$data$SLHcase)
model0.auc

## generate classification plots
model0.class <- classify(model0.pred, model0.mcmc$data$SLHcase, seq(0, 1, by = 0.05))
plot(model0.class, "comp")
```

```
   Min. 1st Qu.  Median    Mean 3rd Qu.    Max. 
 0.8514  0.8518  0.8519  0.8519  0.8521  0.8524 
```

Appendix

Non-centring

To try to improve the mixing we can non-centre some of the parameters. We will focus on non-centring some of the other parameters with respect to the intercept. Therefore, if β_0 is the intercept and β_1 is a regression

parameter, then the current parameterisation is:

$$\log\left(\frac{p_i}{1-p_i}\right) = \beta_0 + \beta_1 x_i,$$
$$\beta_0 \sim N\left(0, 100^2\right),$$
$$\beta_1 \sim N\left(0, 100^2\right).$$

Under the non-centred parameterisation, we instead model β_0 and $\tilde{\beta}_1$, where $\tilde{\beta}_1 = \beta_0 + \beta_1$, resulting in:

$$\log\left(\frac{p_i}{1-p_i}\right) = \beta_0 + \left(\tilde{\beta}_1 - \beta_0\right) x_i,$$
$$\beta_0 \sim N\left(0, 100^2\right),$$
$$\tilde{\beta}_1 \sim N\left(\beta_0, 100^2\right).$$

Hence under the latter parameterisation, updating β_0 conditional on $\tilde{\beta}_1$ also updates β_1 in the likelihood term. We then update the $\tilde{\beta}_1$ terms conditional on β_0 and so on. It is possible to do partial non-centring, however in this case we choose a simpler approach, in which we perform fully centred updates for β_0 and β_1, followed by a fully non-centred update for β_0 given $\tilde{\beta}_1$.

Note that we can also non-centre the random intercepts terms. Under the current parameterisation we have:

$$\log\left(\frac{p_i}{1-p_i}\right) = \beta_0 + \theta_j,$$
$$\beta_0 \sim N\left(0, 100^2\right),$$
$$\theta_j \sim N\left(0, \sigma_\theta^2\right).$$

Under the non-centred parameterisation, we instead model β_0 and $\tilde{\theta}_j$, where $\tilde{\theta}_j = \beta_0 + \theta_j$, resulting in:

$$\log\left(\frac{p_i}{1-p_i}\right) = \beta_0 + \left(\tilde{\theta}_j - \beta_0\right),$$
$$\beta_0 \sim N\left(0, 100^2\right),$$
$$\tilde{\theta}_j \sim N\left(\beta_0, \sigma_\theta^2\right).$$

Hence under the latter parameterisation, updating β_0 conditional on $\tilde{\beta}_1$ also updates β_1 in the likelihood term. We then update the $\tilde{\theta}_j$ terms conditional on β_0 and so on. Again, we perform fully centred updates for β_0 and θ_j, followed by a fully non-centred update for β_0 given $\tilde{\theta}_j$.

Finally, we can also do a similar reparameterisation for the random effect variances. Under the current parameterisation we have:

$$\log\left(\frac{p_i}{1-p_i}\right) = \theta_j,$$
$$\theta_j \sim N\left(0, \sigma_\theta^2\right).$$

Under the non-centred parameterisation, we instead model $\tilde{\theta}_j$ and σ_θ, where $\tilde{\theta}_j = \theta_j / \sigma_\theta$, resulting in:

$$\log\left(\frac{p_i}{1-p_i}\right) = \sigma_\theta \tilde{\theta}_j,$$
$$\tilde{\theta}_j \sim N(0, 1).$$

Hence under the latter parameterisation, updating σ_θ conditional on $\tilde{\theta}_j$ also updates θ_j in the likelihood term. We then update the $\tilde{\theta}_j$ terms conditional on σ_θ and so on. Again, we perform fully centred updates for σ_θ and θ_j, followed by a fully non-centred update for σ_θ given $\tilde{\theta}_j$.

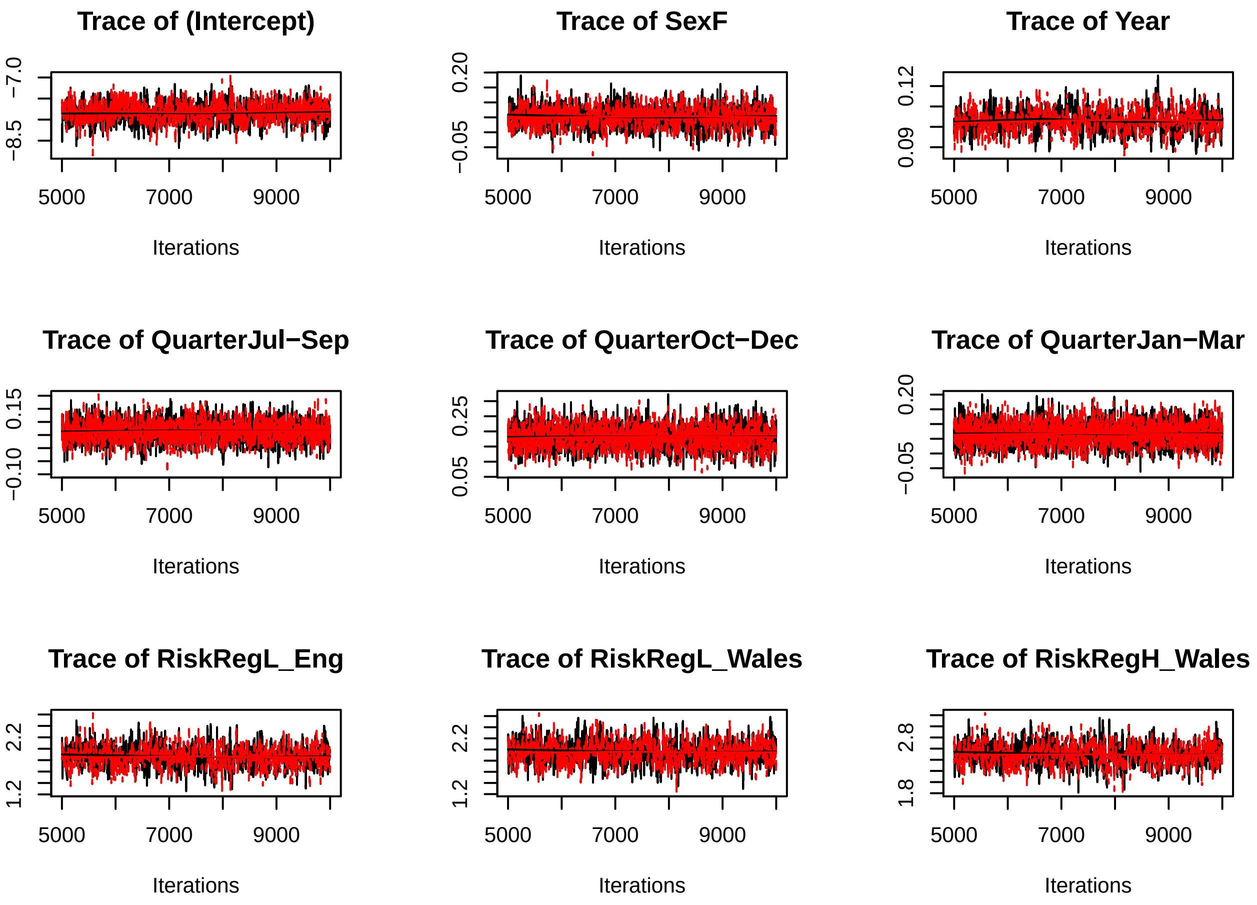

Figure H: Trace plots for regression lines

Trace plots for regression terms for FULL model (not including hierarchical intercepts)

These are shown in Figures H–J:

Binned residual plots for FULL model

As a useful check of the model, we can use the binned residual plots (Figure K) of (Gelman and Hill 2007), implemented in the `arm` package (Gelman and Su 2015):

```
## generate binned residual plot based on posterior
## predictive means
temp_mn <- apply(model.pred$pred, 2, mean)
binnedplot(temp_mn, model.mcmc$data$SLHcase - temp_mn, main = 'Binned residual plot')
rm(temp_mn)
```

Trace plots for regression terms for model without SLH effects (not including hierarchical intercepts)

These are shown in Figures L–N.

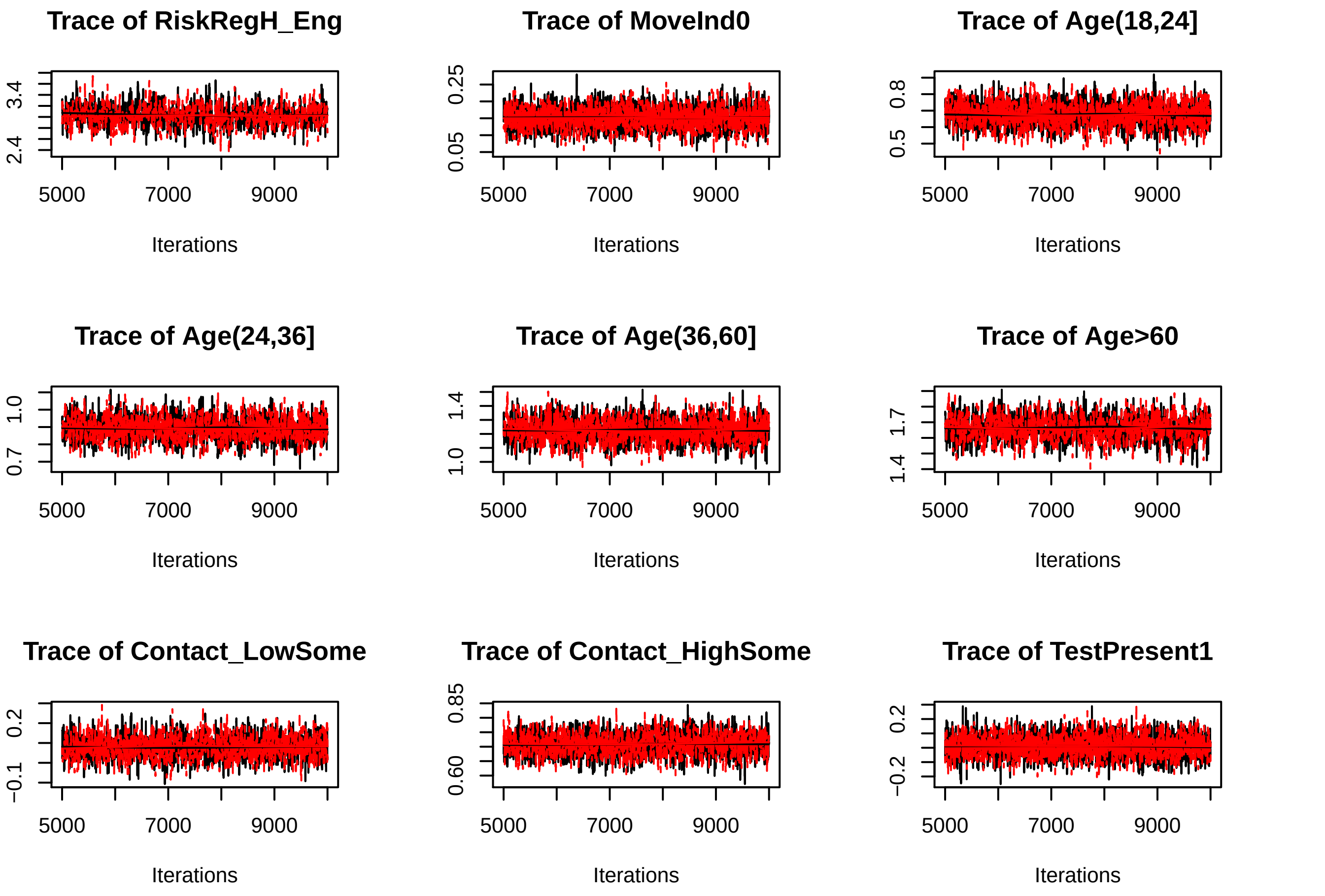

Figure I: Trace plots for regression lines

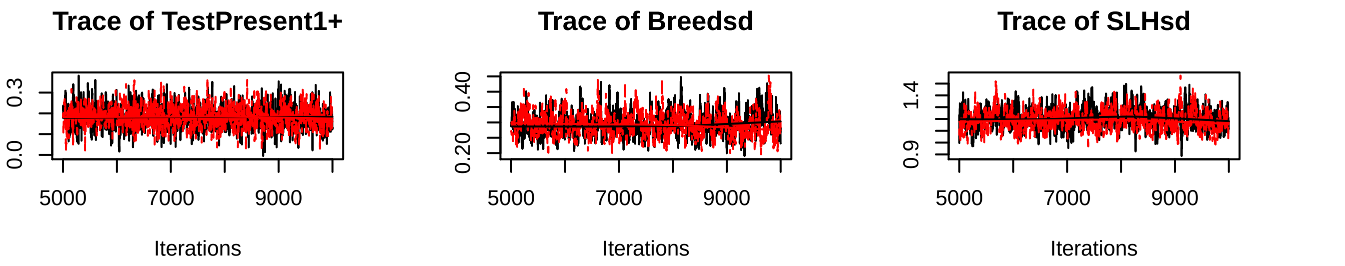

Figure J: Trace plots for regression lines

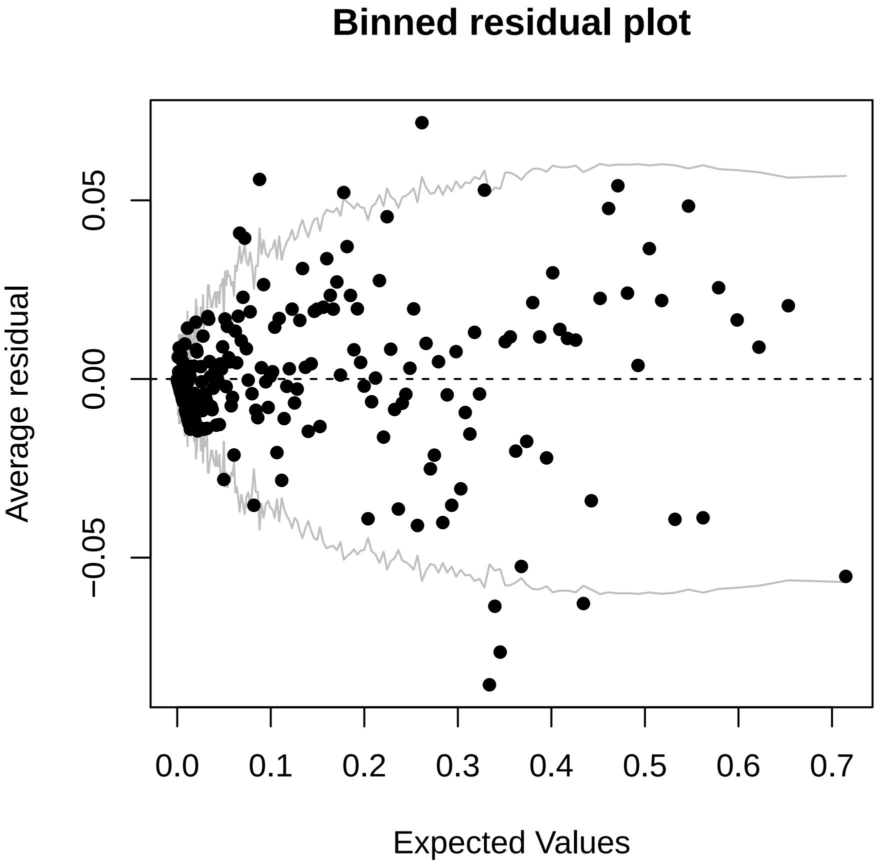

Figure K: Binned residual plots using posterior predictive means as point estimates

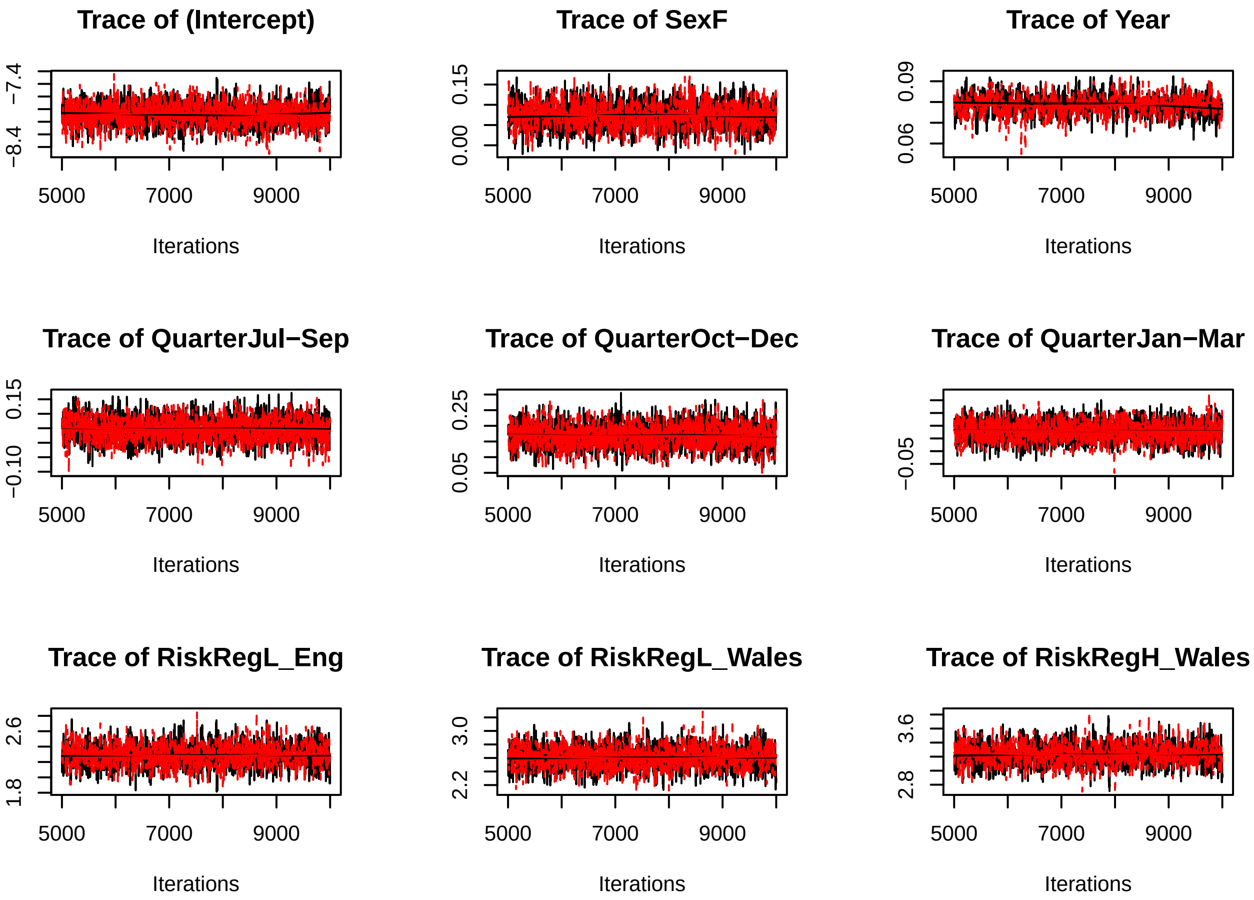

Figure L: Trace plots for regression lines

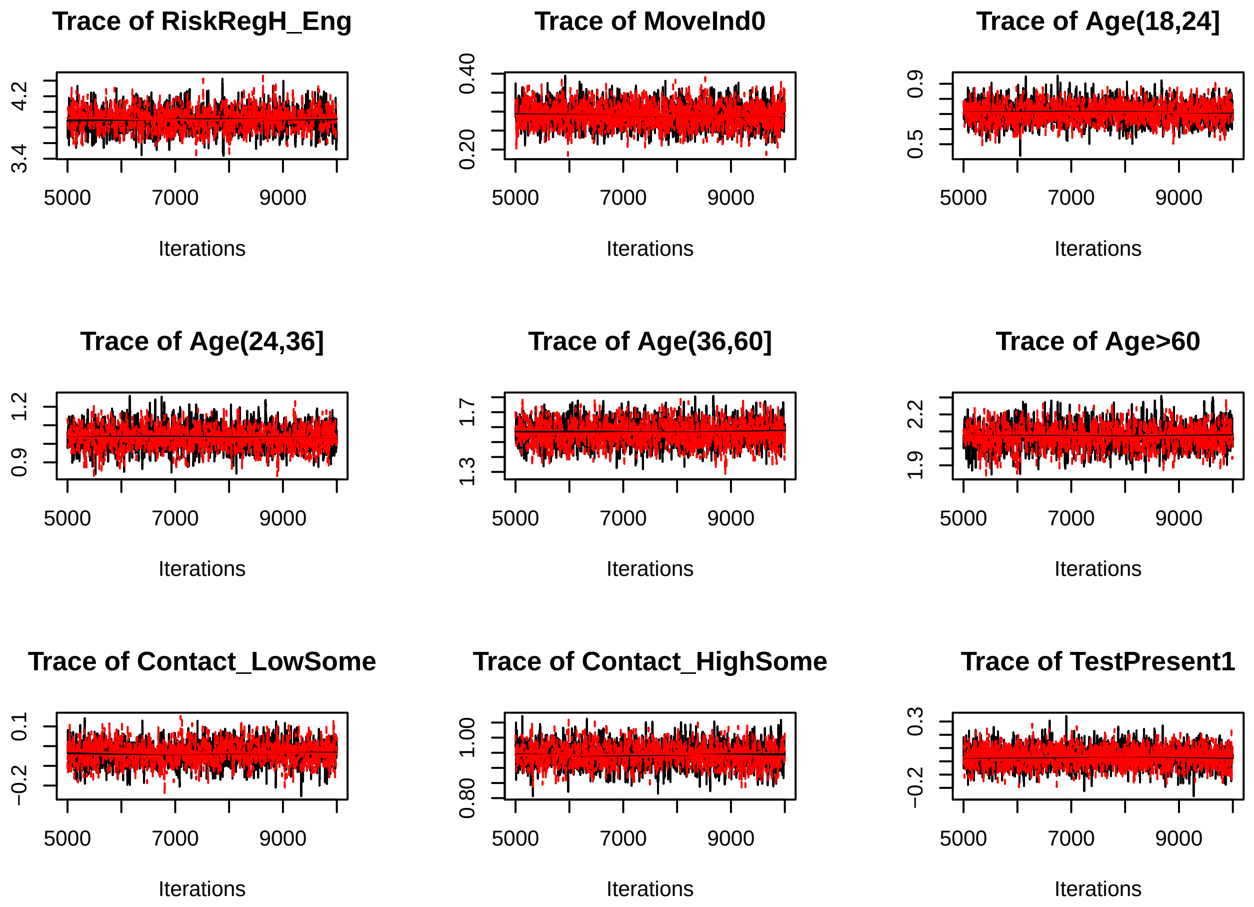

Figure M: Trace plots for regression lines

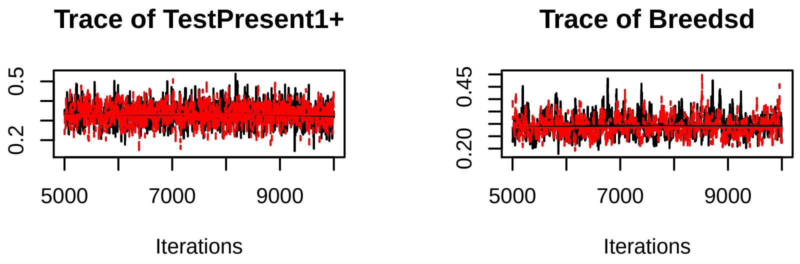

Figure N: Trace plots for regression lines

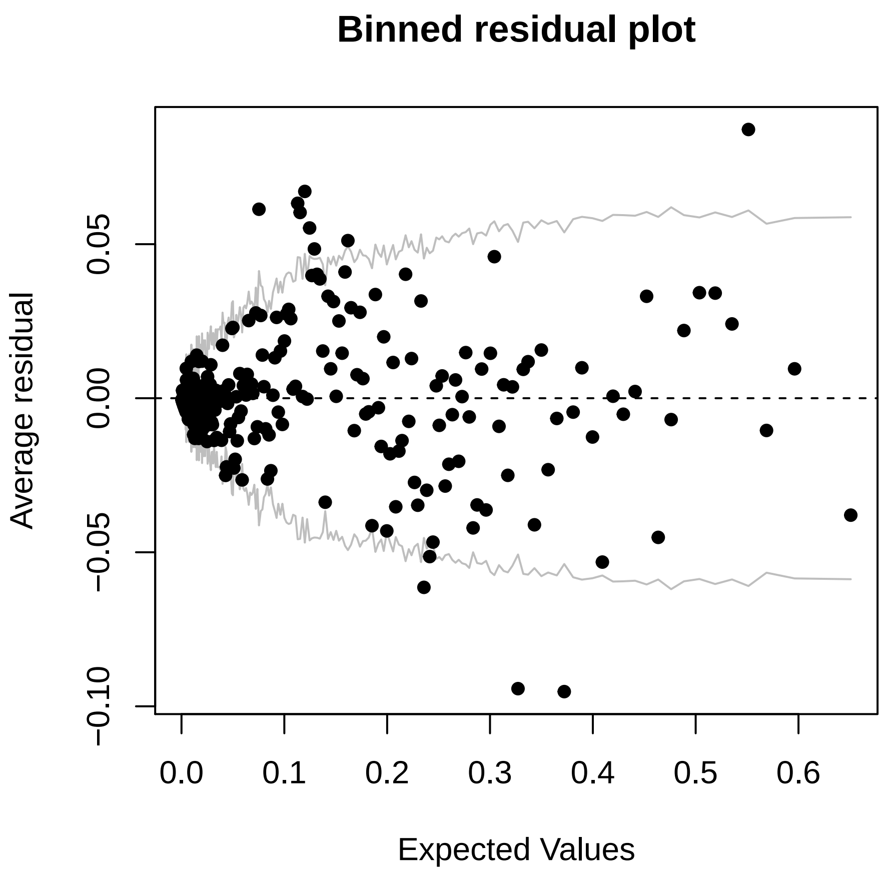

Figure O: Binned residual plots using posterior predictive means as point estimates

Binned residual plots for model without SLH effects

These are shown in Figure O.

```
## generate binned residual plot based on posterior
## predictive means
temp_mn <- apply(model0.pred$pred, 2, mean)
binnedplot(temp_mn, model0.mcmc$data$SLHcase - temp_mn, main = 'Binned residual plot')
rm(temp_mn)
```

References

Allaire, JJ, Joe Cheng, Yihui Xie, Jonathan McPherson, Winston Chang, Jeff Allen, Hadley Wickham, Aron Atkins, and Rob Hyndman. 2016. *Rmarkdown: Dynamic Documents for R*. https://CRAN.R-project.org/package=rmarkdown.

Auguie, Baptiste. 2017. *GridExtra: Miscellaneous Functions for "Grid" Graphics*. https://CRAN.R-project.org/package=gridExtra.

Bates, Douglas, Martin Mächler, Ben Bolker, and Steve Walker. 2015. "Fitting Linear Mixed-Effects Models Using lme4." *Journal of Statistical Software* 67 (1): 1–48. doi:10.18637/jss.v067.i01.

Bivand, Roger, and Nicholas Lewin-Koh. 2016. *Maptools: Tools for Reading and Handling Spatial Objects*.

https://CRAN.R-project.org/package=maptools.

Daróczi, Gergely, and Roman Tsegelskyi. 2015. *Pander: An R Pandoc Writer.* https://CRAN.R-project.org/package=pander.

Eddelbuettel, Dirk. 2013. *Seamless R and C++ Integration with Rcpp.* Springer New York.

Eddelbuettel, Dirk, and Romain Francois. 2011. "Rcpp: Seamless R and C++ Integration." *Journal of Statistical Software* 40 (8): 1–18. http://www.jstatsoft.org/v40/i08/.

Eddelbuettel, Dirk, and Conrad Sanderson. 2014. "RcppArmadillo: Accelerating R with High-Performance C++ Linear Algebra." *Computational Statistics and Data Analysis* 71 (March): 1054–63. http://dx.doi.org/10.1016/j.csda.2013.02.005.

Gelman, Andrew, and Jennifer Hill. 2007. *Data Analysis Using Regression and Multilevel/Hierarchical Models.* Cambridge University Press.

Gelman, Andrew, and Yu-Sung Su. 2015. *Arm: Data Analysis Using Regression and Multilevel/Hierarchical Models.* https://CRAN.R-project.org/package=arm.

Gu, Zuguang, Lei Gu, Roland Eils, Matthias Schlesner, and Benedikt Brors. 2014. "*Circlize* Implements and Enhances Circular Visualization in R." *Bioinformatics* 30 (19): 2811–2. https://cran.r-project.org/package=circlize.

Koohafkan, Michael C. 2015. *Kfigr: Integrated Code Chunk Anchoring and Referencing for R Markdown Documents.* https://CRAN.R-project.org/package=kfigr.

Papaspiliopoulos, Omiros, Gareth O. Roberts, and Martin Sköld. 2003. "Non-Centered Parameterizations for Hierarchical Models and Data Augmentation." In *Bayesian Statistics 7*, edited by J. M. Bernardo, M. J. Bayarri, J. O. Berger, A. P. Dawid, D. Heckerman, A. F. M. Smith, and M. West, 307–26. Oxford University Press.

Plummer, Martyn, Nicky Best, Kate Cowles, and Karen Vines. 2006. "CODA: Convergence Diagnosis and Output Analysis for Mcmc." *R News* 6 (1): 7–11. http://CRAN.R-project.org/doc/Rnews/.

R Core Team. 2016. *R: A Language and Environment for Statistical Computing.* Vienna, Austria: R Foundation for Statistical Computing. https://www.R-project.org/.

RStudio Team. 2015. *RStudio: Integrated Development Environment for R.* Boston, MA: RStudio, Inc. http://www.rstudio.com/.

Tuszynski, Jarek. 2014. *CaTools: Tools: Moving Window Statistics, Gif, Base64, Roc Auc, Etc.* https://CRAN.R-project.org/package=caTools.

Wickham, Hadley. 2007. "Reshaping Data with the reshape Package." *Journal of Statistical Software* 21 (12): 1–20. http://www.jstatsoft.org/v21/i12/.

———. 2009. *Ggplot2: Elegant Graphics for Data Analysis.* Springer-Verlag New York. http://ggplot2.org.

———. 2016. *Scales: Scale Functions for Visualization.* https://CRAN.R-project.org/package=scales.

Wickham, Hadley, and Romain Francois. 2016. *Dplyr: A Grammar of Data Manipulation.* https://CRAN.R-project.org/package=dplyr.

Xie, Yihui. 2015. *Dynamic Documents with R and Knitr.* 2nd ed. Chapman; Hall/CRC.
